# Distinct Assembly Processes Structure Planktonic Bacterial Communities Among Near- and Offshore Ecosystems in the Yangtze River Estuary

**DOI:** 10.1007/s00248-024-02350-x

**Published:** 2024-02-14

**Authors:** Wen-Dong Xian, Junjie Ding, Jinhui Chen, Wu Qu, Pinglin Cao, Chunyu Tang, Xuezhu Liu, Yiying Zhang, Jia-Ling Li, Pandeng Wang, Wen-Jun Li, Jianxin Wang

**Affiliations:** 1 Marine Microorganism Ecological & Application Lab, Zhejiang Ocean University, Haida South Rd No. 1, Dinghai, Zhoushan, 316000 China; 2 State Key Laboratory of Biocontrol, Guangdong Provincial Key Laboratory of Plant Resources and Southern Marine Science and Engineering Guangdong Laboratory (Zhuhai), School of Life Sciences, Sun Yat-Sen University, Guangzhou, 510275 Guangdong China

**Keywords:** Free-living, Particle-attached, Community assembly, Nearshore, Offshore, Yangtze River Estuary

## Abstract

The estuarine system functions as natural filters due to its ability to facilitate material transformation, planktonic bacteria play a crucial role in the cycling of complex nutrients and pollutants within estuaries, and understanding the community composition and assembly therein is crucial for comprehending bacterial ecology within estuaries. Despite extensive investigations into the composition and community assembly of two bacterial fractions (free-living, FLB; particle-attached, PAB), the process by which bacterioplankton communities in these two habitats assemble in the nearshore and offshore zones of estuarine ecosystems remains poorly understood. In this study, we conducted sampling in the Yangtze River Estuary (YRE) to investigate potential variations in the composition and community assembly of FLB and PAB in nearshore and offshore regions. We collected 90 samples of surface, middle, and bottom water from 16 sampling stations and performed 16S rRNA gene amplicon analysis along with environmental factor measurements. The results unveiled that the nearshore communities demonstrated significantly greater species richness and Chao1 indices compared to the offshore communities. In contrast, the nearshore communities had lower values of Shannon and Simpson indices. When compared to the FLB, the PAB exhibit a higher level of biodiversity and abundance. However, no distinct alpha and beta diversity differences were observed between the bottom, middle, and surface water layers. The community assembly analysis indicated that nearshore communities are predominantly shaped by deterministic processes, particularly due to heterogeneous selection of PAB; In contrast, offshore communities are governed more by stochastic processes, largely due to homogenizing dispersal of FLB. Consequently, the findings of this study demonstrate that nearshore and PAB communities exhibit higher levels of species diversity, while stochastic and deterministic processes exert distinct influences on communities among near- and offshore regions. This study further sheds new light on our understanding of the mechanisms governing bacterial communities in estuarine ecosystems.

**Supplementary Information:**

The online version contains supplementary material available at 10.1007/s00248-024-02350-x.

## Introduction

Estuaries are one of the most important marine ecosystems due to their role as the transitional zone between terrestrial rivers and the saline open sea. The high variability of land salinity and nutrient input make estuaries a terrigenous nutrient enriched and ecological sensitive coastal environment [1, 2]. Planktonic bacteria play a crucial role in the cycling of complex matter within estuaries; they transform particulate organic matter (POM) and dissolved organic matter (DOM), derived from ocean and rivers, into biomass that serves as food for microbial food webs and transfer energy and carbon to higher trophic levels [3, 4]. In shallow and turbid estuarine systems, bacterial communities undergo adaptation and specialization driven by sediment resuspension caused by currents, tides, and wind [5, 6]. The sinking of POM is responsible for transporting carbon and nutrients from the surface layer to the deep ocean [7]. During this sinking process, POM is often colonized and simultaneously decomposed by particle-attached (PA) microbes, leading to the release of DOM into the surrounding seawater, which serves as a fuel source for free-living (FL) microbes [8]. Recently, several studies have focused on investigating PA and FL bacterial (PAB and FLB) communities in marine [9–12], lacustrine [13, 14], and estuarine environments [15, 16]. Comparing PAB and FLB communities can yield valuable insights into the potential interactions between the two communities and the difference in ecological processes present within them [17, 18].

PAB and FLB communities exhibit differences in taxonomic composition, physiological metabolism, lifestyle, and ecological behavior [10, 19, 20]. For example, PAB are larger and occur in higher local concentrations than FLB in water [19]. Bacterial carbon production measurements have shown significantly higher activity for PAB compared to FLB in both freshwater and estuarine samples [20]; In terms of taxonomy, *Proteobacteria* were found to be the most abundant microbes in the PAB community, whereas *Actinobacteria* dominated the FLB community [10]. Due to their smaller size, FLB are more easily dispersed compared to PAB [13]. The ecological and oceanographic processes drive the response of ocean microbiomes to environmental changes [21]; there is a lack of knowledge regarding the community assembly processes of FLB and PAB communities in estuarine ecosystems, specifically between nearshore and offshore regions. In the community assembly theory, deterministic processes refer to environmental selection, including environmental filtering and interactions among species (competition, predation, and facilitation). Stochastic processes refer to dispersal and ecological drift, the dispersal means the movement of species across space, speciation is the generation of new genetic variation, and ecological drift represents random changes in species’ relative abundance over time due to the inherent [22]. The estuary is a transitional zone between land and ocean interactions, with significant changes in salinity and abundant habitats, making it a breeding ground for many marine fish; microbial activity strongly influences these organisms [23]. It is of both theoretical and practical significance to elucidate the community assembly of estuarine microorganisms.

The Yangtze River, also known as the Changjiang River, is the third longest river in the world and receives a substantial amount of nutrients from its basin [24]. Our previous study in Yangtze River Estuary (YRE) have shown that PAB community are less affected by environmental filtration, while homogeneous selection and drift were important processes in the FLB community assembly [16], However, geographical comparisons are lacking. In this study, 16 sampling stations were selected at the Yangtze River Estuary across near- and offshore regions, and water samples from the surface, middle, and bottom layers were collected. The differences in community structure and assembly between FLB and PAB in nearshore and offshore regions were determined using 16S rRNA gene amplicon sequencing and the neutral community model (NCM) analysis [25]. Community assembly is simultaneously influenced by factors that are relatively deterministic and factors that are more stochastic. The deterministic class includes selection imposed by the abiotic environment and both antagonistic and synergistic species interactions. The stochastic class includes unpredictable disturbance, probabilistic dispersal and random birth-death events [26]. This study aims to provide insights into (1) bacterial structures and diversity between the FLB and PAB communities and (2) bacterial community assembly process within the nearshore and offshore regions of the YRE.

## Materials and Methods

### Sample Collection and Environmental Factor Measurement

**Fig. 1:**
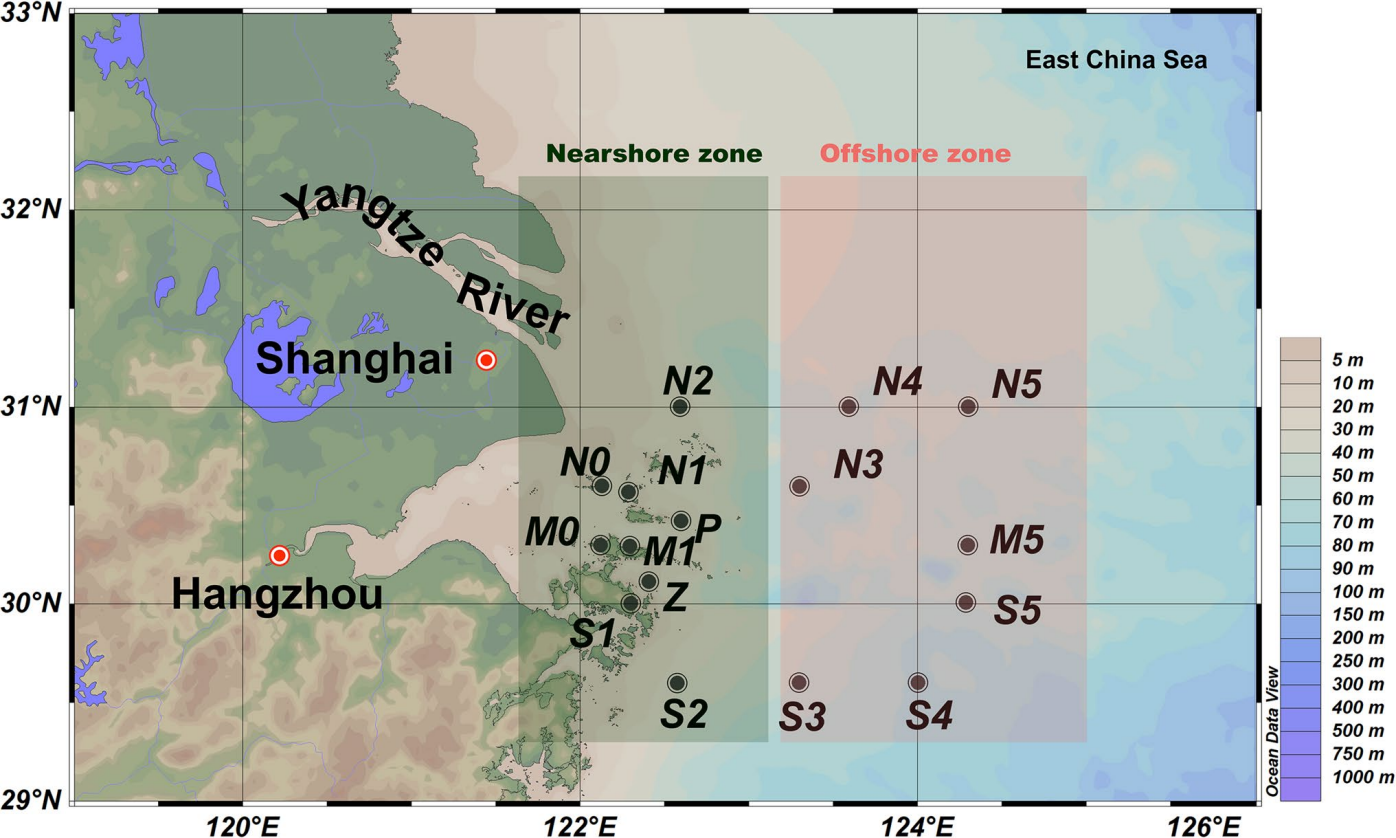
An overview of geographical location in the Yangtze River Estuary (dark circles). A total of 90 samples were collected from 16 sites in October 2020. The map was generated with the Ocean Data View (http://odv.awi.de); water depths are indicated with colored column on the right side. Stations belong to near- or offshore region were shown in light green and light red boxes, respectively

Seawater samples were collected from 16 sampling stations along the YRE in October 2020, using conductivity-temperature-depth sensors (Sea-Bird Electronics SBE 32) and Go-Flo® bottles (Fig. 1). Samples were collected from the surface, middle, and bottom of the water columns, with the middle samples missing from stations N0, N1, and M0 due to limited water depth (<20 m). To obtain particle-attached (PA) and free-living (FL), microbial cells were sequentially filtered using 3-μm and 0.22-μm membrane filters (Millipore Corporation, Billerica, MA, USA) under mild vacuum pressure (<33.3 kPa). All samples were immediately stored at −20 °C on board and subsequently transferred to −80 °C in the laboratory for DNA extraction. In total, 90 samples were collected and categorized into four groups: surface layer (~1-m depth), middle layer (20-m depth), bottom layer (water layer above sediment), nearshore (<50 m)/offshore (>50 m) and FL/PA fractions.

Environmental variables, including temperature, salinity, depth, pH, chlorophyll a (Chla), suspended solids (SS), total alkalinity (ALK), chemical oxygen demand (COD), and dissolved oxygen (DO), were measured on-site using a CTD system. ammonia nitrogen (NH_4_^+^), nitrite (NO_2_^−^), silicate (SiO_4_^4−^) and phosphorus (PO_4_^3−^) were analyzed using the SmartChem automatic nutrient analyzer (Smartchem 200, Alliance, France); nitrate (NO_3_^−^) was determined using the copper-cadmium reduction method following the procedure described by Sorte and Basak [27], All physical and chemical parameters adhere to the Marine Monitoring Specification (GB17378, 2007). To gain insights into the influence of water flow on bacterial communities, information on ocean currents at different depths (1 m, 20 m, and 50 m) was also collected using the CTD system.

### DNA Extraction, PCR, and Sequencing

DNA from water samples was extracted using the PowerSoil DNA Isolation Kit (MO BIO, San Diego, CA, USA), following the kit instructions. DNA quality was assessed using 1% agarose gel electrophoresis and the Nanodrop 2000 (Thermo, Waltham, MA, USA). For bacterial 16S rRNA gene amplification, 515F and 806R primers were utilized. The reaction mixture consisted of 4 μL FastPfu buffer, 2 μL dNTPs (2.5 mM), 0.80 μL forward primers (5 μM), 0.80 μL reverse primers (5 μM), 0.40 μL FastPfu polymerase, 0.20 μL bovine serum albumin (BSA), 10 ng DNA, and double-distilled H2O, with a total volume of 20 μL. Amplification reactions were conducted using an ABI GeneAmp® 9700 PCR instrument (ABI, Waltham, MA, USA). The PCR products were used to construct paired-end libraries following the manufacturer's instructions. High-throughput sequencing was performed by Majorbio Bio-Pharm Technology Co., Ltd. (Shanghai, China) on the Illumina MiSeq platform.

### Amplicon Analysis

Raw reads were processed and analyzed using the QIIME2 platform [28]. Primer excision and quality control were performed using VSEARCH [29]. ASV (amplicon sequence variant) clustering was conducted by calling unoise3 in USEARCH [29], Taxonomy annotations for each ASV were performed using the RDP classifier [30] against the Silva database (v138) [31]. ASVs annotated as “Chloroplast,” “Eukaryote,” “Archaea,” and “Mitochondria” were filtered out. ASVs table was generated by mapping primers-removed reads to the representative sequences of ASVs. To account for sequencing depth, the ASVs table was rarefied based on the lowest reads number (12,259) of all samples using the “rarefy” function of vegan package [32] (Fig. S1).

### Statistical Analysis

All statistical analyses were performed using R v4.0 [33]. Bacterial diversity was calculated as the Shannon, Chao1, Simpson, and Richness indices using the picante package [34]. The statistically significant difference in alpha diversity between FLB and PAB was analyzed using the two-sided Wilcoxon signed-rank test from the ggsignif package [35] and plotted with the ggplot2 package [36]. A principal coordinate analysis (PCoA) analysis was conducted using the vegan package, the dissimilarity matrix was calculated by the Bray-Curtis dissimilarity method, *R*^2^ and *p* value was also calculated, the *R*^2^ indicates the variance can be explained by geographical distance, the *p* value indicates the significance of the variance [37]. Full linkage hierarchical clustering based on Bray-Curtis dissimilarity was performed using the vegan package [32]. Non-metric multidimensional scaling (NMDS) analysis was also performed using the vegan and ggplot2 packages [38]. Mantel test analysis was conducted using the vegan package [39]. Heatmaps were generated using the pheatmap package to visualize the Spearman correlations between environmental factors and the top 30 phyla [40]. The vegan and randomForest packages were used to calculate and visualize the contribution of biomarker species among nearshore and offshore communities [41]. Venn diagram were generated using the UpSetR package [42].

### Specificity-Occupancy Plots

The term “specificity” refers to the distribution of microbes exclusively within certain communities, while “occupancy” refers to the broad distribution range of microbes. In order to examine the distribution of ASVs across sampling sites within the same habitat and their specificity to that habitat, we calculated the occupancy and specificity of individual ASVs for each habitat and projected them onto maps [43]. The ASVs with a relative abundance greater than 1% were selected from the ASV table for each habitat, and from the table, specificity and occupancy were calculated in Jiang et.al [43]. In our study, specificity is defined by the number of individual ASVs (*S*) in the samples of a habitat (*H*) (Eq. 1), and occupancy is defined by the relative frequency of occurrence of *S* in the samples of *H* (Eq. 2).

1 $$\text{Specificity}=\frac{\text{N}-{\text{individuals}}_{\;\mathrm S,\,\mathrm H}}{\text{N}-{\text{individuals}}_{\;\mathrm S}}$$

2 $$\text{Occupancy}=\frac{\text{N}-{\mathrm{sites}}_{\;\mathrm S,\,\mathrm H}}{\text{N}-\mathrm{Sites}\;_{\mathrm H}}$$

N-individual _S,H_ is the mean number of individual ASVs across all samples in habitat *H*, while N-individual _S_ is the sum of the mean number of individual *S* over all habitats; N-sites _S,H_ is the number of samples in *H* where *S* is present, while N-sites _H_ is the total number of samples in *H* [44]. These two metrics were then used as the axes in the specificity-occupancy plots. To find specialist taxa attributable to each habitat type, ASVs with specificity and occupancy greater or equal to 0.7 was selected according to Gweon’s method [45].

### Community Assembly Analysis

The Stegen’s null model based analyses [26] were conducted using the iCAMP package [46]. This analysis was based on the β-nearest taxon index (βNTI) and the Bray-Curtis-based Raup-Crick index (RC_bray_) [46]. Values of |βNTI| > 1.96 indicate determinism, which can be further classified as homogeneous selection (HoS, βNTI < −1.96) or heterogeneous selection (HeS, βNTI > 1.96). Conversely, values of |βNTI| ≤ 1.96 suggest stochasticity. Integrating the RC_bray_ value, the community assembly processes can be categorized as homogenizing dispersal (HD, RC_bray_ < −0.95), dispersal limitation (DL, RC_bray_ > +0.95), or drift and others processes (with |RC_bray_| < 0.95) representing weak selection, weak dispersal, diversification, and drift processes. The null community was randomized 999 times to obtain average null expectations [47]. The neutral community model (NCM) was also fitted using the Hmisc package to assess the contributions of stochastic processes in shaping FL and PA communities [48].

## Results

### Overview of Water Properties

A total of 13 environmental factors including salinity, temperature, SS, DO, NO_2_^−^, NO_3_^−^, PO_4_^3−^, NH_4_^+^, COD, SiO_4_^4−^, pH, total alkalinity, and Chla were measured (Table S1). The values for these factors are as follows: salinity ranged from 21.96 to 34.50 g/L, SS ranged from 0.60 to 534.70 mg/L, temperature ranged from 13.95 to 20.81 °C, COD ranged from 0.02 to 2.81 mg/L, DO ranged from 6.78 to 8.51 mg/L, Chla ranged from 0.05 to 0.35 μg/L, NO_2_^−^ranged from 0.0004 to 0.004 mg/L, NH_4_^+^ ranged from 0.001 to 0.012 mg/L, NO_3_^−^ ranged from 0.01 to 0.48 mg/L, PO_4_^3−^ ranged from 0.002 to 0.02 mg/L, SiO_4_^4−^ ranged from 0.06 to 1.49 mg/L, and pH ranged from 8.06 to 8.24. The alkalinity values were in the range of 21.96 to 34.50 g/L. Additionally, ocean currents were measured at different depths (1 m, 20 m, and 50 m) (Fig. S2). In the YRE, the water forms an eddy, and the velocity increases from the surface to the bottom, ranging from 0.1 to 0.2 m/s.

### Community Structure and Diversity of FL and PA Bacteria

A total of 6,117,739 high-quality bacterial sequences were obtained from the 90 samples, revealing the presence of 51 phyla, 320 orders, and 758 genera in the seawater of the YRE. Among these taxa, phyla including *Proteobacteria*, SAR324, *Actinobacteriota*, *Bacteroidota*, *Marinimicrobia*, *Dadabacteria*, *Chloroflexi*, *Gemmatimonadota*, *Nitrospinota*, *Cyanobacteria*, and *Verrucomicrobiota* were shared across all samples, indicating their widespread presence. Additionally, *Margulisbacteria* was exclusively detected in offshore samples, while *Desulfobacterota* was only found in the nearshore region. The phylum PAUC34f exhibited distribution in both nearshore and offshore environments.

**Fig. 2:**
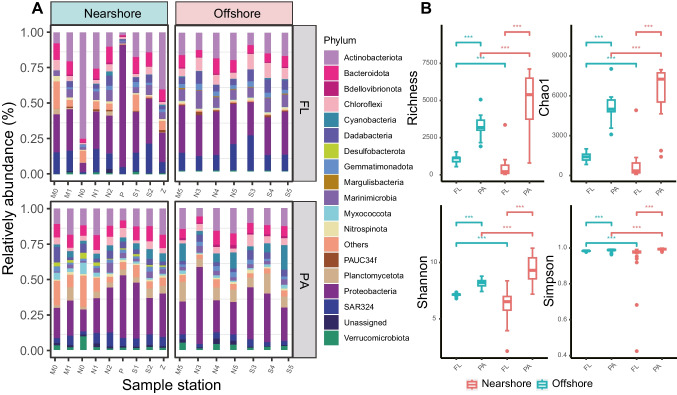
Dominant bacterioplankton composition and alpha diversity between FL and PA bacterial communities among near- and offshore zones. **A** ASVs with Relative abundance of sequences ≥ 2% was calculated, the top ten phyla for each sample were used, others represent the phyla not unassigned at the phylum level. **B** Richness, Chao1, Shannon, and Simpson index. *p* values of Tukey’s HSD (honestly significant difference) test between groups are indicated on the top of bar plots. ***p < 0.001

The distribution of microbial taxa in the surface, middle, and bottom layers showed similar patterns. *Proteobacteria*, *Actinobacteriota*, and *Bacteroidota* were consistently dominant phyla across all three layers, with *Proteobacteria* exhibiting particularly high relative abundance (57.33%) at station P. Other abundant taxa included SAR324, *Chloroflexi*, *Marinimicrobia*, *Planctomycetota*, *Dadabacteria*, *Cyanobacteria*, and *Gemmatimonadota* (Fig. S3). However, notable differences were observed between the FLB and PAB communities. For instance, *Planctomycetota* (7.56%) displaying a significant advantage in the PAB community, but only 0.14% in the FLB community (Fig. 2). On the other hand, *Gemmatimonadota* (0.03%), *Nitrospinota* (1.19%), and PAUC34f (0.31%) exhibited greater dominance in the FLB community compared to only 0.01%, 0.38%, and 0.03% in the PAB community (Fig. 2), *Cyanobacteria* (1.6%) and *Verrucomicrobiota* (2.1%) showed higher relative abundance in the PAB community, but only 0.15%, 0.61% in the FLB community. Furthermore, PAUC34f (0.31%) and *Margulisbacteria* (0.30%) were exclusively detected in the FLB communities, while *Desulfobacterota* (0.36%) was found solely in PAB communities. *Proteobacteria* and *Actinobacteriota* were the two most abundant phyla in both the FLB and PAB communities. Subtle variations between the FLB and PAB communities among sample stations were also observed. For instance, in the FLB communities, *Actinobacteriota* (77.54%) exhibited the highest relative abundance at the N0 site, while *Planctomycetota* (3.50%) dominated at the P2 site. In the PAB communities, *Alphaproteobacteria* (29.02%) displayed the highest relative abundance at the N3 site. Random forest analysis was employed to identify microbial biomarkers at the ASV level, ASVs belongs to *Desulfobacterota*, *Myxococcota*, *Acidobacteriota*, and *Cyanobacteria* are biomarkers for offshore communities, while *Planctomycetota Firmicutes* SAR324 and *Marinimicrobia* are characteristic phyla in nearshore regions (Fig. S4).

**Fig. 3:**
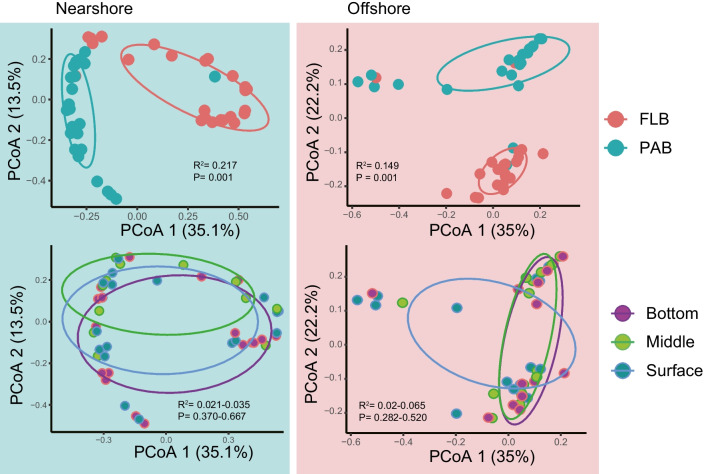
Principal coordinate analysis (PCoA) of bacterioplankton communities based on Bray–Curtis dissimilarity. Communities within ellipses are associated with 95% confidence intervals. The red, green, pink, light green, and dark blue circles represent FLB (free-living bacterial communities), PAB (particle-attached bacterial communities), bottom, middle, and surface water, respectively. Samples within green and red boxes are bacterial communities of near- and offshore, respectively. *R*^2^ indicates the variance can be explained by geographical distance, the *p* value indicates the significance of the variance

Significant differences on alpha diversity were observed between near- and offshore (*p* < 0.001, Fig. 2B). In comparison to the offshore communities, the nearshore communities exhibited significantly higher species richness and Chao1 indices, while the Shannon and Simpson indices were lower. The four diversity indices of PAB community were higher than FLB community, indicating greater diversity and richness of the PAB community (Fig. 2B). However, no significant differences (*p* > 0.05) were found between the surface layer, middle layer, and bottom layer (Fig. S5). These community differences were also supported by PCoA analysis. The nearshore communities showed distinct separation between the FAB and PAB communities (*R*^2^ = 0.217, *p* = 0.01) compared to the offshore communities (*R*^2^ = 0.14, *p* = 0.001) (Fig. 3). No significant differences in beta-diversity were found between the three layers (*R*^2^ = 0.20–0.065, *p* > 0.05). The NMDS analysis further confirmed these findings (Fig. S6).

### Effects of Environmental Factors on Bacterial Communities

The Mantel test was conducted to analyze the influence of environmental factors on bacterial community composition (Table 1). For the nearshore communities, several environmental factors showed significant effects (*p* < 0.01), including COD, DO, SS, PO_4_^3−^, SiO_4_^4−^, and NO_3_^−^. Conversely, in offshore communities, significant effects were observed for DO, Chla, temperature, and salinity. pH, Alk, NO_2_^−^, and NH_4_^+^ had no discernible influence on both nearshore and offshore communities. In summary, the nearshore community was primarily influenced by COD, SS, PO_4_^3−^, SiO_4_^4−^, and NO_3_^−^, whereas offshore communities were most influenced by Chla, temperature, and salinity. Among these factors, Salinity (*r* = 0.21) exerted the strongest influence on the offshore communities, while SiO_4_^4−^ (*r* = 0.26) had the most pronounced impact on the nearshore communities. The correlation analysis between environmental factors and bacterial taxa at the phylum level was also conducted on different lifestyles (FLB and PAB) (Fig. S7). In addition to SS, other environmental factors have varying degrees of influence on bacterial taxa in FLB; moreover, Chla, temperature, DO, NO_2_^−^, and NH_4_^+^ present a significantly correlation (*p* < 0.05) with almost all taxa in FLB. However, there are no environmental factor shows universal and significant (*p* < 0.05) correlation with these taxa (Fig. S7B).

**Table 1:** Correlations between the environmental factors and the bacterial community (Mantel test)

Environmental factors	Nearshore		Offshore	
	*r*	*p*	*r*	*p*
COD	0.2515	0.003**	0.05726	0.242
DO	0.2802	0.001***	0.2049	0.003***
pH	0.04665	0.283	0.1726	0.055
Alk	0.08086	0.198	0.09865	0.077
Chla	0.1526	0.053	0.2656	0.001***
SS	0.2437	0.008**	-0.07094	0.709
PO_4_^3−^	0.1199	0.009**	0.01225	0.4
SiO_4_^4-^	0.209	0.001***	0.03919	0.261
NO_2_^−^	−0.09713	0.939	−0.05378	0.775
NH_4_^+^	0.05146	0.238	0.00488	0.336
NO_3_^−^	0.2596	0.002**	0.04966	0.248
Temperature	0.02837	0.342	0.1909	0.002**
Salinity	0.09351	0.142	0.2081	0.007**

<0.05, *; <0.01, **; <0.001, ***

### Occupancy and Specificity of ASVs in the Near- and Offshore Regions

**Fig. 4:**
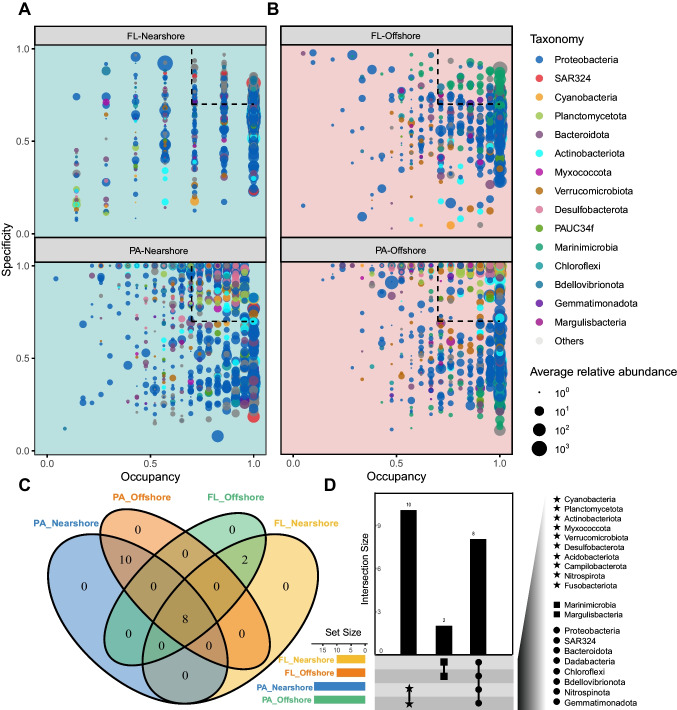
Specificity-occupancy plots showing ASVs that different between the PA and FL in the nearshore and offshore water from the Yangtze River Estuary. **A**, **B** The x-axis represents occupancy, i.e., how well an ASV is distributed in each habitat across all sites; the y-axis represents specificity, i.e., whether ASVs are also found in other habitats. **C** Venn diagrams showing the numbers of unique and shared ASVs among the four bacterial communities. **D** Detailed bacterial phyla of unique and shared ASVs. Dotted box on the top right are ASVs having a spec-occ > 0.7

To investigate the distribution of different ASVs across sampling stations within the same regions (nearshore and offshore) and their specificity to the regions, the occupancy and specificity of individual ASVs were calculated. To identify specialist taxa associated with each lifestyle and region, ASVs with a specificity and occupancy of 0.7 or greater (indicated by dotted boxes in Fig. 4A, B) were examined. specificity-occupancy analysis indicates that the occupancy rate of ASVs originating from nearshore exhibits greater variability, whereas the occupancy rate of ASVs originating from offshore remains relatively consistent (Fig. 4A, B). Since ASVs related to *Cyanobacteria*, *Planctomycetota*, *Actinobacteriota*, *Verrucomicrobiota*, *Desulfobacterota* and PAUC34f were mainly found in PAB, while ASVs of *Marinimicrobia* and SAR324 mainly lives in FLB, the higher occupancy of nearshore regions is attributed to the absence of *Marinimicrobia*, which enhances specificity (Fig. 4A, B). ASVs with a specificity and occupancy of 0.7 or greater of each lifestyle showed varying occupancy but were commonly found in most offshore sites (Fig. 4B). Furthermore, overall ASVs were more frequent and prevalent in offshore habitats (Fig. 4B). These differences in ASVs shared by nearshore and offshore were due to a combination of different occurrence frequencies and habitat selections of the ASVs. *Proteobacteria*, SAR324 (Marine group B), *Bacteroidota*, *Dadabacteria*, *Chloroflexi*, *Bdellovibrionota*, *Nitrospinota*, and *Gemmatimonadota* were found in all four specialist groups: FLB-nearshore, PAB-nearshore, FLB-offshore, PAB-offshore (Fig. 4D). Notably, *Planctomycetota* (7.56%) displaying a significant advantage in the PAB community, but only 0.14% in the FLB community (Fig. 2). On the other hand, *Gemmatimonadota *(0.03%), *Nitrospinota *(1.19%), and PAUC34f (0.31%) exhibited greater dominance in the FLB community compared to only 0.01%, 0.38%, and 0.03% in the PAB community (Fig. 2). Importantly, the result of the Venn diagram shows no specialists were identified between the nearshore and offshore communities (Fig. 4D).

### Microbial Community Assembly Mechanisms in Near- and Offshore Regions

**Fig. 5:**
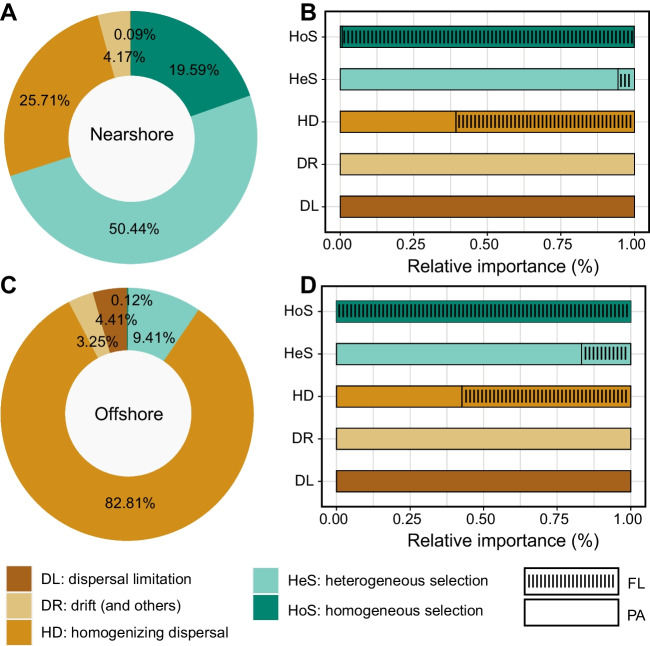
Relative importance of different ecological processes between near- and offshore communities. **A, B** Nearshore. **C, D** Offshore. Values on the ring indicate the fraction of ecological processes (deterministic: homogeneous and heterogeneous selection; stochastic: dispersal limitations and homogenizing dispersal; drift and others).

**Fig. 6:**
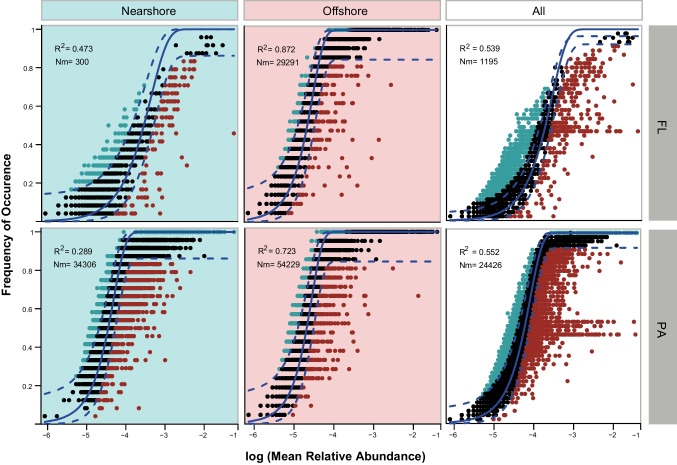
Fit of the neutral community model (NCM) of community assembly. The predicted occurrence frequencies for nearshore, offshore, and all represent bacterial communities from nearshore (green colored), offshore (pink colored), and both region (white colored), respectively. The solid blue lines indicate the best fit to the NCM as in Sloan et al., and the dashed blue lines represent 95% confidence intervals around the model prediction. ASVs that occur more or less frequently than predicted by the NCM are shown in green or red colors. Nm represents the metacommunity size multiplied by immigration, and *R*^2^ represents the fit to the model

The null model based analyses were employed to explore the relative contributions of deterministic and stochastic processes to community assembly in nearshore and offshore bacterial communities. βNTI confirmed the contribution of determinism community assembly processes in the nearshore communities and stochastic processes among offshore bacterial communities (Fig. S8). The large proportion of heterogeneous selection (50.44%) and homogeneous selection (19.59%) suggested that deterministic processes exerted greater influence on nearshore community (Fig. 5A). In turn, the relative high proportion of homogenizing dispersal (82.81%) in the offshore communities indicated that stochastic processes played greater role in governing community assembly (Fig. 5C). FLB communities were mainly influenced by heterogeneous selection, while PAB communities were mainly influenced by homogenizing dispersal and drift (Fig. 5B, D) across all regions. The NCM analysis successfully captured a significant portion of the relationship between the occurrence frequency of ASVs and their mean relative abundances (Fig. 6). For FLB communities, the NCM explained 47.30%, 87.20%, and 53.90% of the community variance in the nearshore, offshore, and both regions, respectively. Similarly, for PAB communities, the NCM explained 28.90%, 72.30%, and 55.20% of the community variance in the nearshore, offshore, and both regions, respectively. Moreover, the NCM analysis revealed a higher explained community variance in the offshore planktonic community (ranging from 72.30 to 87.20%, with a mean value of 79.75%) compared to the nearshore community (ranging from 28.90 to 47.30%, with a mean value of 38.10%). Additionally, the Nm-value, representing species dispersal, was higher for bacterial taxa in the offshore regions (Nm = 29,291–54,229) compared to the nearshore region (Nm = 300–34,306) (Fig. 6), which indicate that the dispersal of planktonic bacteria species is higher in offshore environments than in nearshore regions. Both the null model and NCM analysis suggested that the nearshore communities are shaped by deterministic processes and offshore communities are governed by stochastic processes.

## Discussion

The Yangtze River basin supports a dense population and well-developed industry and agriculture. The YRE, adjacent to the Zhoushan fishing grounds, serves as a traditional fishing area [49], also a representative estuarine ecosystem. Recent studies have extensively investigated the bacterial compositions and community characteristics in this ecosystem [16, 50–52]. Our study aimed to announce a comprehensive understanding of community assembly of the free-living and particle-attached bacterioplankton by surveying 16 sampling stations in the YRE and its adjacent waters. The results unveiled significant differences among nearshore, offshore, FLB and PAB communities, but no distinct differences were observed between the bottom, middle, and surface water layers (mainly α diversity and β diversity).

### Distinct Microbial Communities Between Near- and Offshore Regions

Despite the alpha diversity being similar across different water depths, distinct microbial communities were observed between nearshore and offshore regions of the YRE. This can be attributed to the enrichment of heterotrophic bacteria resulting from nutrient-rich river inputs in the nearshore region, whereas the relatively oligotrophic offshore environment limits the growth of these microbes [53]. In contrast to previous research [54], no significant differences in diversity were observed among different layers in this study. The frequent mixing of water may be the primary factor contributing to this phenomenon, the in situ current pattern in the YRE region, including the south China Sea surface current, the China coastal current, and the Kuroshio branch current, contributes to water dynamics from the bottom to the surface (Fig. S2) [55], promoting efficient mixing of bacterioplankton in the water column, the high dissolved oxygen (DO) values (>6 mg/L, Table S1) also indicate the efficient mixing in the YRE during sampling [56].

The higher richness and diversity of bacterial species in PAB community has been observed in open ocean environments [57], the number of ASVs in the PAB community is also found significantly higher than that in the FLB community. The particulate organic matter (POM) serves as a nutrient source for PAB, and their higher capacity for rapid decomposition and utilization of organic carbon may explain this phenomenon [58, 59]. We found that *Margulisbacteria* was exclusively present in offshore samples, while *Desulfobacterota* was detected solely in the nearshore region (Fig. 2, Fig. 6D), suggesting potential environmental preferences. *Margulisbacteria* has been reported to specifically attach to spirochetes and thrive in ocean waters [60, 61], while *Desulfobacterota* prefers nutrient-rich environments [62] and not sensitive to contamination [63]. In consistent with previous studies [64], *Planctomycetota* shows a closer association with particle-attached (PA) samples (Fig. 2), This may be due to the preference of *Planctomycetota* microbes for an anaerobic environment, and the organic aggregates developed by PAB provide an anaerobic microenvironment [65]. Previous studies have revealed that *Verrucomicrobia* was widely distributed across the marine envrionment [66]; our results further demonstrated that *Verrucomicrobia* was more dominant in the nearshore community than in offshore. The random forest analysis also identified *Verrucomicrobia* as an important indicator taxon for the nearshore community (Fig. S4). Fluctuations in the abundance of *Verrucomicrobia* in particle-attached bacterial communities have been reported in several Arctic studies [67]; the preference of *Verrucomicrobia* for organic matter-rich marine environments [68] and the abundance of organic matter in nearshore waters due to terrigenous input [69] could be the underlying reason.

### Environmental Factors Have Different Influences on Near- and Offshore Communities

The nearshore communities were primarily influenced by COD, SS, PO_4_^3−^, SiO_4_^4−^and NO_3_^−^, while offshore communities were mainly influenced by Chla, temperature, and salinity. Among these factors, salinity (*r* = 0.208) and Si (*r* = 0.259) exhibit the strongest influences on offshore and nearshore communities, respectively. Salinity gradient has been identified as a crucial impact factor on bacterioplankton in estuary ecosystems [70]. SS, carried by river inflow, supplies a large amount of nutrients, could be due to the enrichment of microbial communities in nearshore region, which has been frequently reported [71]. Further investigation is needed to determine the impacts of other environmental factors on nearshore and offshore bacterial communities. Numerous studies have shown significant correlations between PAB and FLB and environmental parameters [15, 72]. The distinction between these two communities is primarily attributed to the different microhabitats they inhabit in aquatic ecosystems. Attached bacteria reside on particle surfaces, while FLB float freely in the water column. Previous studies have indicated that FLB bacteria are more sensitive to environmental variables compared to PAB [15, 73]. In our study, the Chla, temperature, DO, NO_2_^−^, and NH_4_^+^ significantly influences (*p* < 0.05) the majority of phylum-level taxa in FLB, while only a limited number of taxa are affected by detected environmental factors, which also demonstrate that a higher proportion of free-living variation can be explained by environmental parameters (as shown in the heatmap, Fig. S7) compared to PAB. This can be attributed to the fact that PAB attached to particles act as “buffers” or micro-islands [73, 74], while FL cells are directly exposed to the surrounding water. Therefore, particle size plays a crucial role, as larger particles to which bacteria are attached make them less sensitive to changes in the surrounding water [73]. Additionally, the high bacterial density on particles facilitates efficient signaling and quorum sensing, quorum sensing signals, such as acylated homoserine lactones, which are required mainly when the population reaches high densities [75], and the nutrient ratio within particles may differ significantly from that in the surrounding seawater. These factors collectively explain why PAB are less influenced by changes in environmental parameters compared to FLB.

### Within Habitat Specificity and Overlap Was Observed Across the Spatial Scale

Planktonic communities in dynamic water columns are generally considered to be “heterogeneous” in both space and time [76]. Despite our study did not take time variation into consideration, this heterogeneous was also observed across the spatial scale. Compared with the nearshore habitats, both FLB and PAB communities in offshore showed higher specificity (Fig. 4B) across the sampling stations, which indicate that the offshore environments are more selective for a large and stable core community. The constant mixing of water from terrestrial rivers and dynamic fluctuations in salinity and nutrient levels within estuarine ecosystems provide ample opportunities for microbial communities to exchange between diverse habitats. The presence of this phenomenon suggests that microorganisms in these aquatic ecosystems possess the capability to adapt different lifestyle in order to optimize nutrient acquisition from the surrounding environment [77]. As shown in the specificity-occupancy plots (Fig. 4), we found that the majority of the ASVs, both FLB and PAB, have high occupancy in YRE, which particularly evident in offshore communities, meaning that most of them are also found in all other sites across the estuarine ecosystem. This may because of the continuous input of fresh water and nutrient from Yangtze River make up a rapidly changing environment and make residents lives there present the rapid exchange among habitats. The mechanisms underlying the spatial homogeneity observed in both near- and offshore communities remain unclear at present. Our study did not explore the physical and chemical characteristics of the PAB and FLB themselves, thus further investigation is needed to determine whether these factors contribute to stabilizing nutrient levels in the overlying water column, thereby fostering similar community structures. Alternatively, it is possible that other factors associated with an attached lifestyle play a role.

### Bacterial Community Assembly Process Influenced by Randomness

Homogeneous selection refers to stable environments where few phylogenetic shifts occur due to consistent abiotic and biotic conditions, whereas heterogeneous selection results from fluctuating environments causing higher phylogenetic diversity [26, 78]. Dispersal is categorized into homogenizing dispersal and dispersal limitation. High dispersal homogenizes communities leading to minor taxonomic variation; while limited dispersal increases taxonomic diversity. If neither dominates, “undominated” or “drift” governs community formation [26, 78]. Analyses based on null model suggested the deterministic and stochastic processes shaped the bacterial communities in nearshore and offshore region, respectively (Fig. 5A, C), which is consistent with the result of NCM analysis (Fig. 6). The results could be due to the relatively unrestricted dispersal is in offshore region [79], where hydrological mixing may increase dispersal-related processes and ecological drift [80, 81]. The NCM analysis explained community variance for PAB and FLB is 53.9% and 55.2%, respectively. This is consistent with the conclusion of our previous study [16], which indicates that both FLB and PAB communities are shaped by stochastic processes in the YRE region. However, a detailed analysis of the near- and offshore regions reveals distinct results. For FLB, 47.30% and 87.20% of the explained community variance is observed in the nearshore and offshore regions, respectively, whereas for PAB, 28.90% and 72.30% of the explained community variance is observed in the nearshore and offshore regions, respectively. This suggests that PAB community in the nearshore are impacted by a stronger effect of deterministic processes, while FLB community in the offshore region are significantly impacted by stochastic processes. Further evidences were provided by the results of null model analysis. Although, PAB in nearshore are predominantly governed by HeS, DR, and DL, compared with HeS (50.44%) and DR (4.17%), the HeS exerts the paramount influence, accounting for 44.14% of the total impact on PAB. Seminally, among the HD (56.93%), HeS (1.65%), and HoS (0.12%) in offshore region, the HeS strongly shapes the assembly of FLB (Fig. 5). The YRE is eutrophic [82], and the availability of nutrients for planktonic microbes makes it compatible with nutrient-rich substrates. PAB abundances are commonly high at the more locations along the nutrient-rich coastal zones [83], high nutrient levels in nearshore regions could be impose a strong selective pressure by selecting for copiotrophs; however, there is more randomness in more oligotrophic waters in offshore region because of a relaxed selective pressure. which may explain why the PAB community is shaped by deterministic processes in the nearshore region and FLB community is predominantly shape by stochastic processes in the offshore region. In this study, FLB in nearshore and offshore have distinct assembly pattern, and the same situation was also observed in PAB; further investigation is needed to determine this phenomenon.

## Conclusion

The present investigation offers an extensive characterization of the PAB and FLB communities in the nearshore and offshore regions of the YRE, encompassing their biodiversity, community structure, the influence of environmental factors, and microbial community assembly mechanisms. Distinct from the FLB community, the PAB community exhibits a higher alpha diversity. In contrast to the offshore region, the nearshore communities display a significantly elevated species richness and Chao1 indices. *Margulisbacteria* was exclusively identified in offshore samples, whereas *Desulfobacterota* was solely detected in the nearshore area*. Planctomycetota* demonstrates a notable advantage within the PAB community, yet *Nitrospinota* exhibits greater predominance in the FLB community. The nearshore communities are significantly influenced by COD, DO, SS, PO_4_^3−^, SiO_4_^4−^, and NO_3_^−^, while the offshore communities are mainly affected by DO, Chla, temperature, and salinity. In the nearshore region, deterministic processes play a vital role in shaping bacterial community assembly, whereas the dispersal of planktonic bacteria species is more prominent in offshore regions.

## Supplementary Information

ESM 1 (PDF 128 kb)

ESM 2 (PDF 4424 kb)

ESM 3 (PDF 214 kb)

ESM 4 (PDF 1312 kb)

ESM 5 (PDF 391 kb)

ESM 6 (PDF 691 kb)

ESM 7 (PDF 833 kb)

ESM 8 (PDF 343 kb)

ESM 9 (CSV 5 kb)

## Data Availability

All sequence data generated in this study have been submitted to the NCBI Sequence Read Archive under the accession number PRJNA877030.
