# Supplementary material for: Distinct Assembly Processes Structure Planktonic Bacterial Communities Among Near- and Offshore Ecosystems in the Yangtze River Estuary
Source: Microb Ecol. 2024 Feb 14;87:42. doi: 10.1007/s00248-024-02350-x (PMC11385042; doi:10.1007/s00248-024-02350-x)
Supplement: Supplementary file 1 [file 248_2024_2350_MOESM1_ESM.pdf]

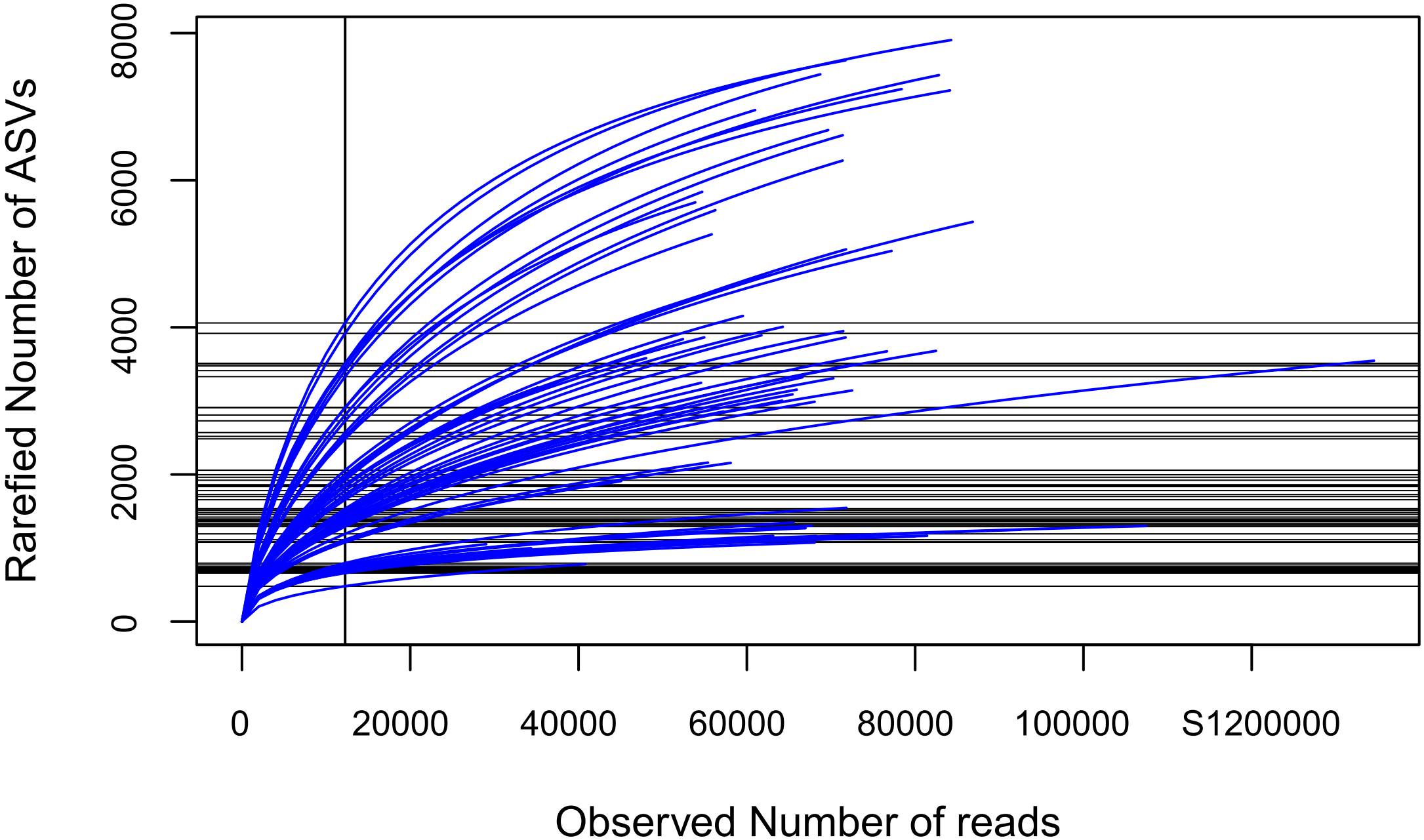

Rarefied Noumber of ASVs
0
2000
4000
6000
8000
0
20000
40000
60000
80000
100000
S1200000
Observed Number of reads
