# Supplementary material for: Distinct Assembly Processes Structure Planktonic Bacterial Communities Among Near- and Offshore Ecosystems in the Yangtze River Estuary
Source: Microb Ecol. 2024 Feb 14;87:42. doi: 10.1007/s00248-024-02350-x (PMC11385042; doi:10.1007/s00248-024-02350-x)
Supplement: Supplementary file 2 [file 248_2024_2350_MOESM2_ESM.pdf]

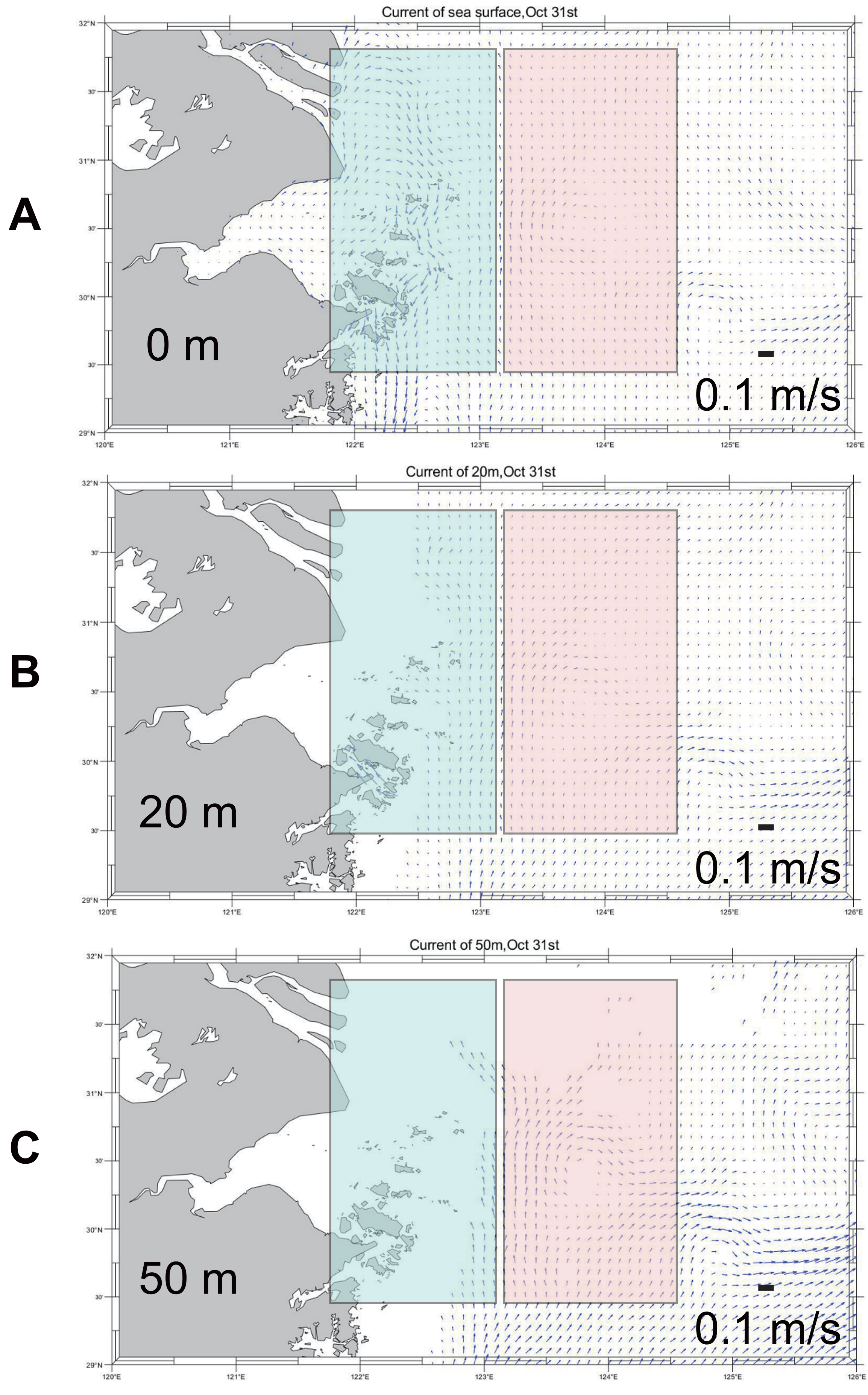
A
Current of sea surface,Oct 31st
0 m
0.1 m/s
B
Current of 20m,Oct 31st
20 m
0.1 m/s
C
Current of 50m,Oct 31st
50 m
0.1 m/s
32°N
30'
31°N
30'
30°N
30'
29°N
120°E
121°E
122°E
123°E
124°E
125°E
126°E
