# Supplementary material for: Distinct Assembly Processes Structure Planktonic Bacterial Communities Among Near- and Offshore Ecosystems in the Yangtze River Estuary
Source: Microb Ecol. 2024 Feb 14;87:42. doi: 10.1007/s00248-024-02350-x (PMC11385042; doi:10.1007/s00248-024-02350-x)
Supplement: Supplementary file 3 [file 248_2024_2350_MOESM3_ESM.pdf]

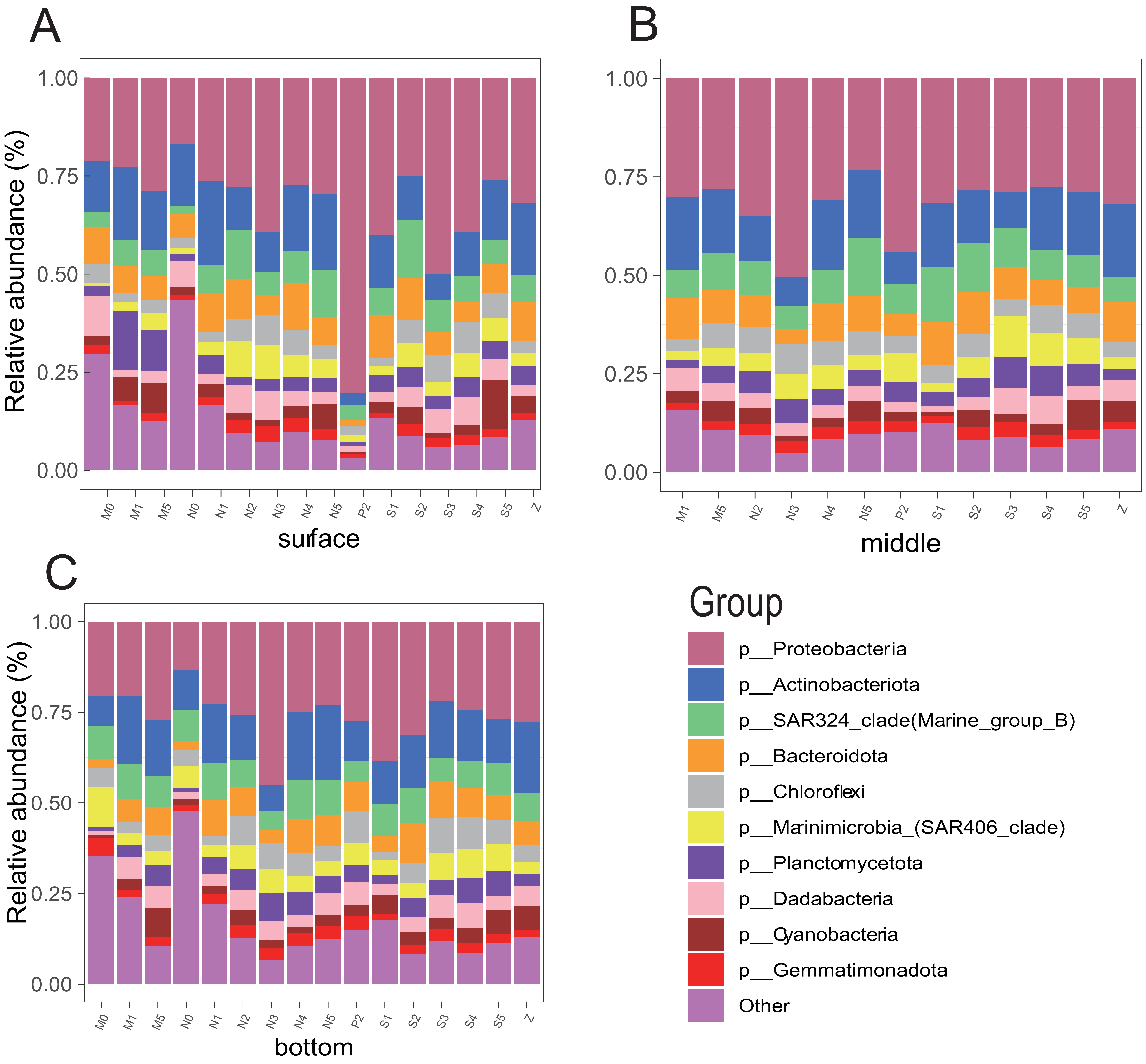
A
B
C
Relative abundance (%)
1.00
0.75
0.50
0.25
0.00
M0
M1
M5
N0
N1
N2
N3
N4
N5
P2
S1
S2
S3
S4
S5
Z
surface
middle
bottom
Group
p__Proteobacteria
p__Actinobacteriota
p__SAR324_clade(Marine_group_B)
p__Bacteroidota
p__Chloroflexi
p__Marinimicrobia_(SAR406_clade)
p__Planctomycetota
p__Dadabacteria
p__Cyanobacteria
p__Gemmatimonadota
Other
