# Supplementary material for: Distinct Assembly Processes Structure Planktonic Bacterial Communities Among Near- and Offshore Ecosystems in the Yangtze River Estuary
Source: Microb Ecol. 2024 Feb 14;87:42. doi: 10.1007/s00248-024-02350-x (PMC11385042; doi:10.1007/s00248-024-02350-x)
Supplement: Supplementary file 4 [file 248_2024_2350_MOESM4_ESM.pdf]

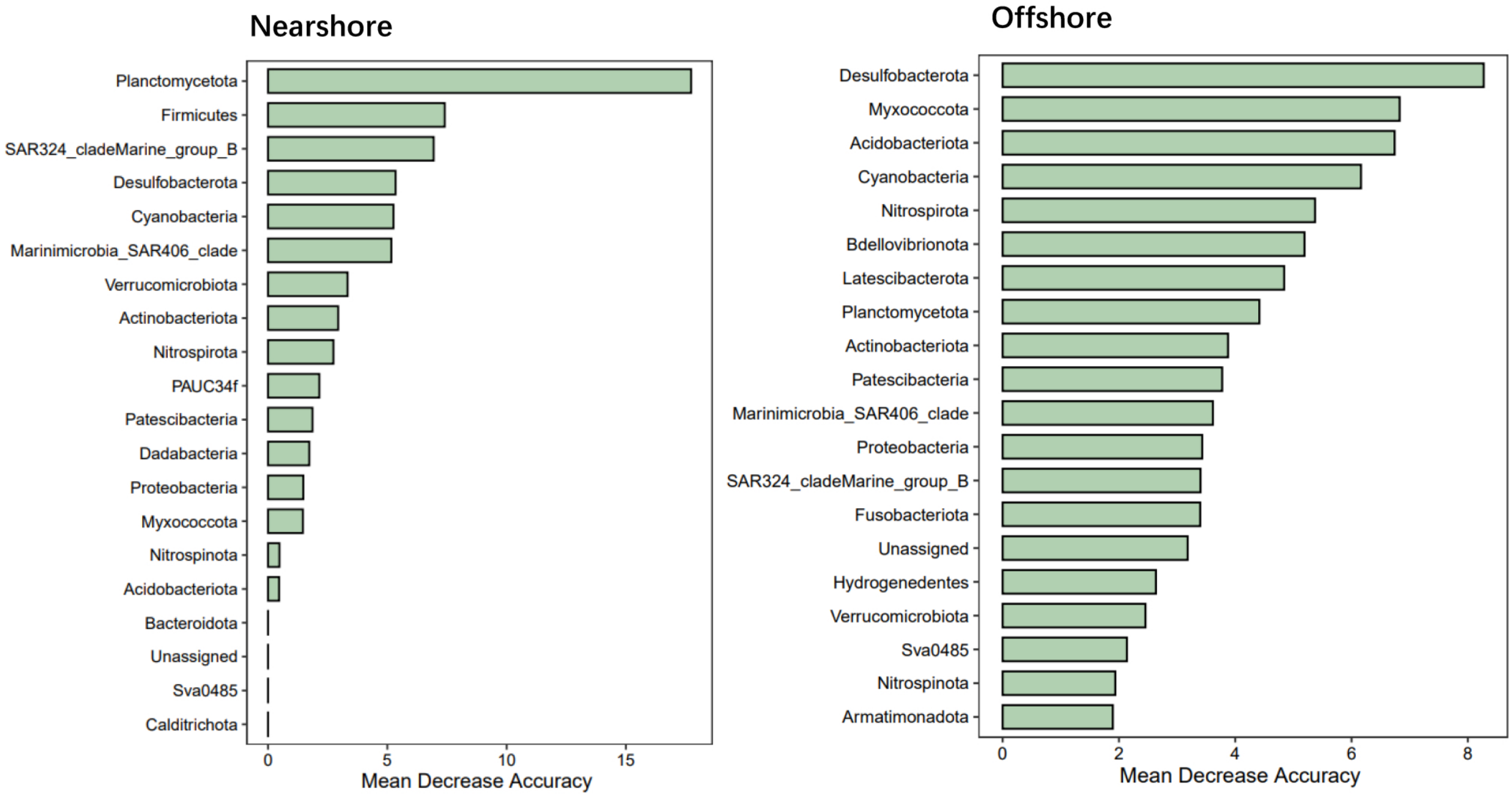
Nearshore
Planctomycetota
Firmicutes
SAR324_cladeMarine_group_B
Desulfobacterota
Cyanobacteria
Marinimicrobia_SAR406_clade
Verrucomicrobiota
Actinobacteriota
Nitrospirota
PAUC34f
Patescibacteria
Dadabacteria
Proteobacteria
Myxococcota
Nitrospinota
Acidobacteriota
Bacteroidota
Unassigned
Sva0485
Calditrichota
0
5
10
15
Mean Decrease Accuracy
Offshore
Desulfobacterota
Myxococcota
Acidobacteriota
Cyanobacteria
Nitrospirota
Bdellovibrionota
Latescibacterota
Planctomycetota
Actinobacteriota
Patescibacteria
Marinimicrobia_SAR406_clade
Proteobacteria
SAR324_cladeMarine_group_B
Fusobacteriota
Unassigned
Hydrogenedentes
Verrucomicrobiota
Sva0485
Nitrospinota
Armatimonadota
0
2
4
6
8
Mean Decrease Accuracy
