# Supplementary material for: Distinct Assembly Processes Structure Planktonic Bacterial Communities Among Near- and Offshore Ecosystems in the Yangtze River Estuary
Source: Microb Ecol. 2024 Feb 14;87:42. doi: 10.1007/s00248-024-02350-x (PMC11385042; doi:10.1007/s00248-024-02350-x)
Supplement: Supplementary file 5 [file 248_2024_2350_MOESM5_ESM.pdf]

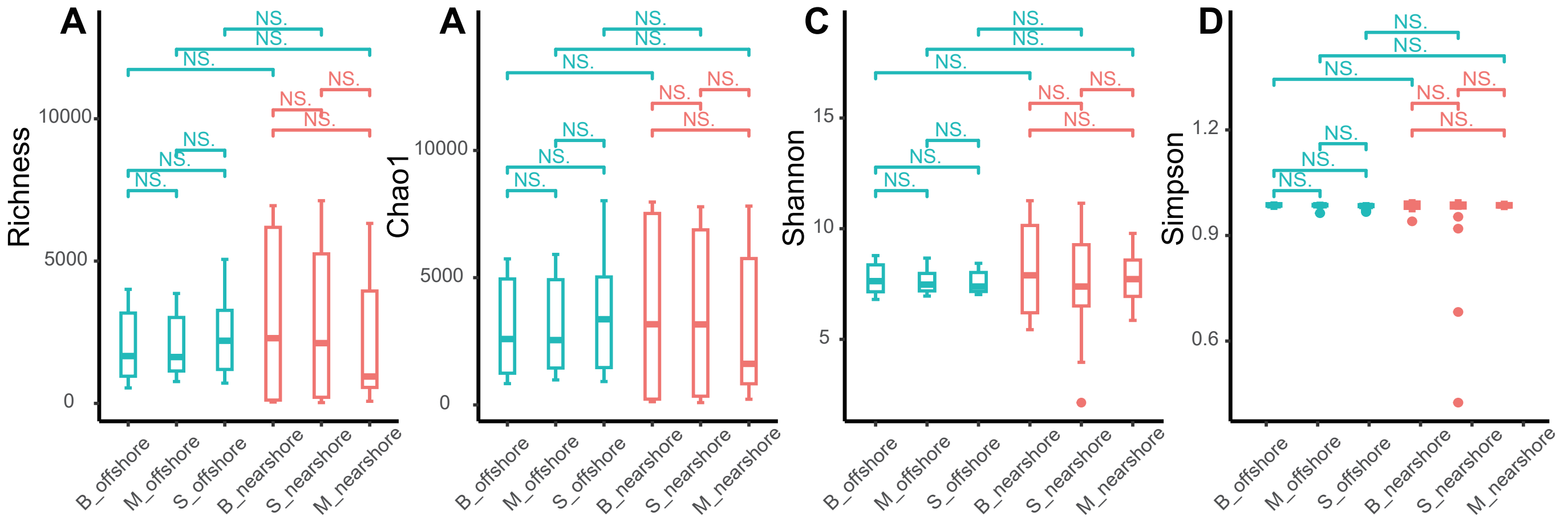
A
Richness
NS.
0
5000
10000
B_offshore
M_offshore
S_offshore
B_nearshore
S_nearshore
M_nearshore
A
Chao1
0
5000
10000
C
Shannon
5
10
15
D
Simpson
0.6
0.9
1.2
