# Supplementary material for: Distinct Assembly Processes Structure Planktonic Bacterial Communities Among Near- and Offshore Ecosystems in the Yangtze River Estuary
Source: Microb Ecol. 2024 Feb 14;87:42. doi: 10.1007/s00248-024-02350-x (PMC11385042; doi:10.1007/s00248-024-02350-x)
Supplement: Supplementary file 6 [file 248_2024_2350_MOESM6_ESM.pdf]

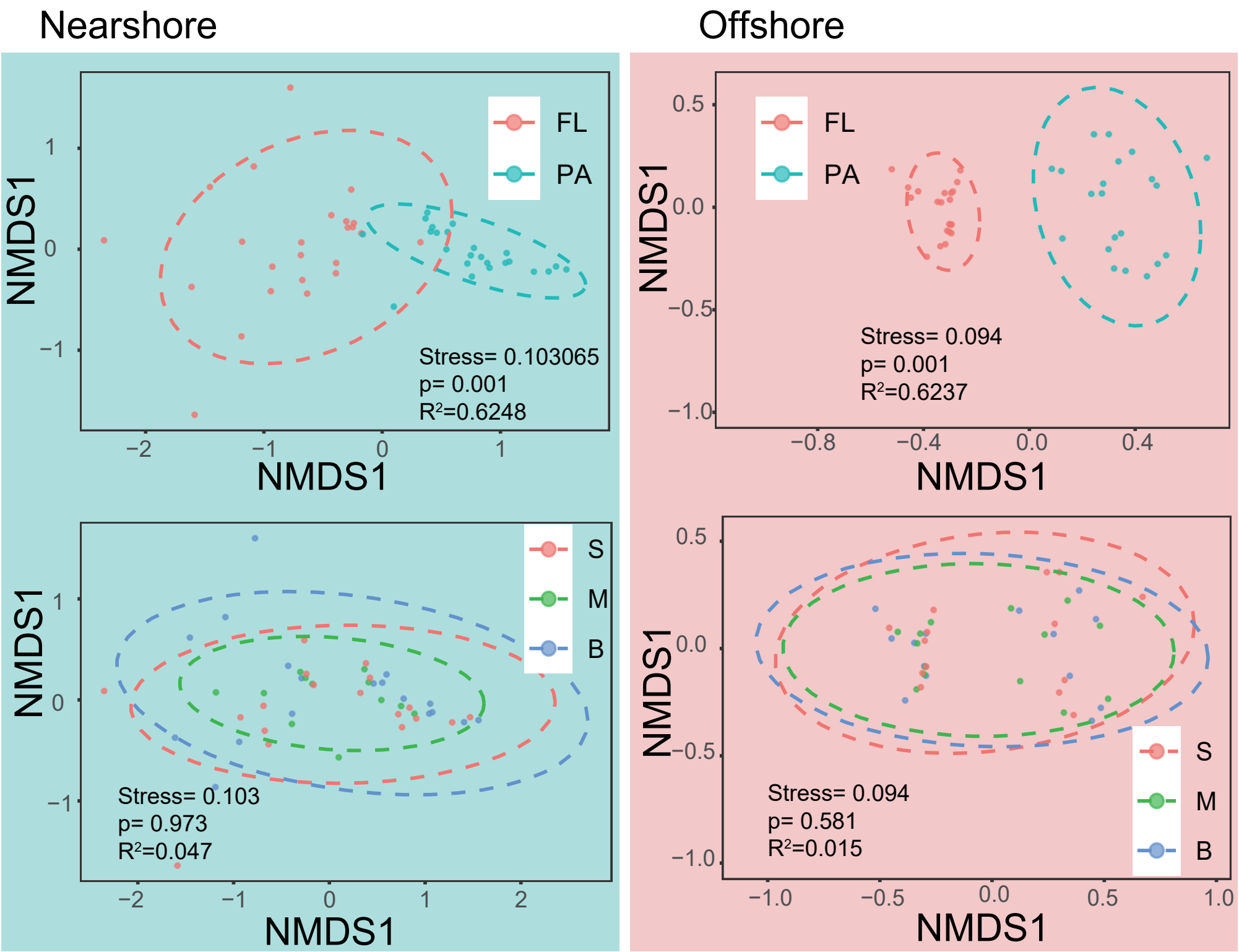
Nearshore
Offshore
FL
PA
Stress= 0.103065
p= 0.001
R²=0.6248
NMDS1
Stress= 0.094
p= 0.001
R²=0.6237
S
M
B
Stress= 0.103
p= 0.973
R²=0.047
Stress= 0.094
p= 0.581
R²=0.015
