# Supplementary material for: Distinct Assembly Processes Structure Planktonic Bacterial Communities Among Near- and Offshore Ecosystems in the Yangtze River Estuary
Source: Microb Ecol. 2024 Feb 14;87:42. doi: 10.1007/s00248-024-02350-x (PMC11385042; doi:10.1007/s00248-024-02350-x)
Supplement: Supplementary file 7 [file 248_2024_2350_MOESM7_ESM.pdf]

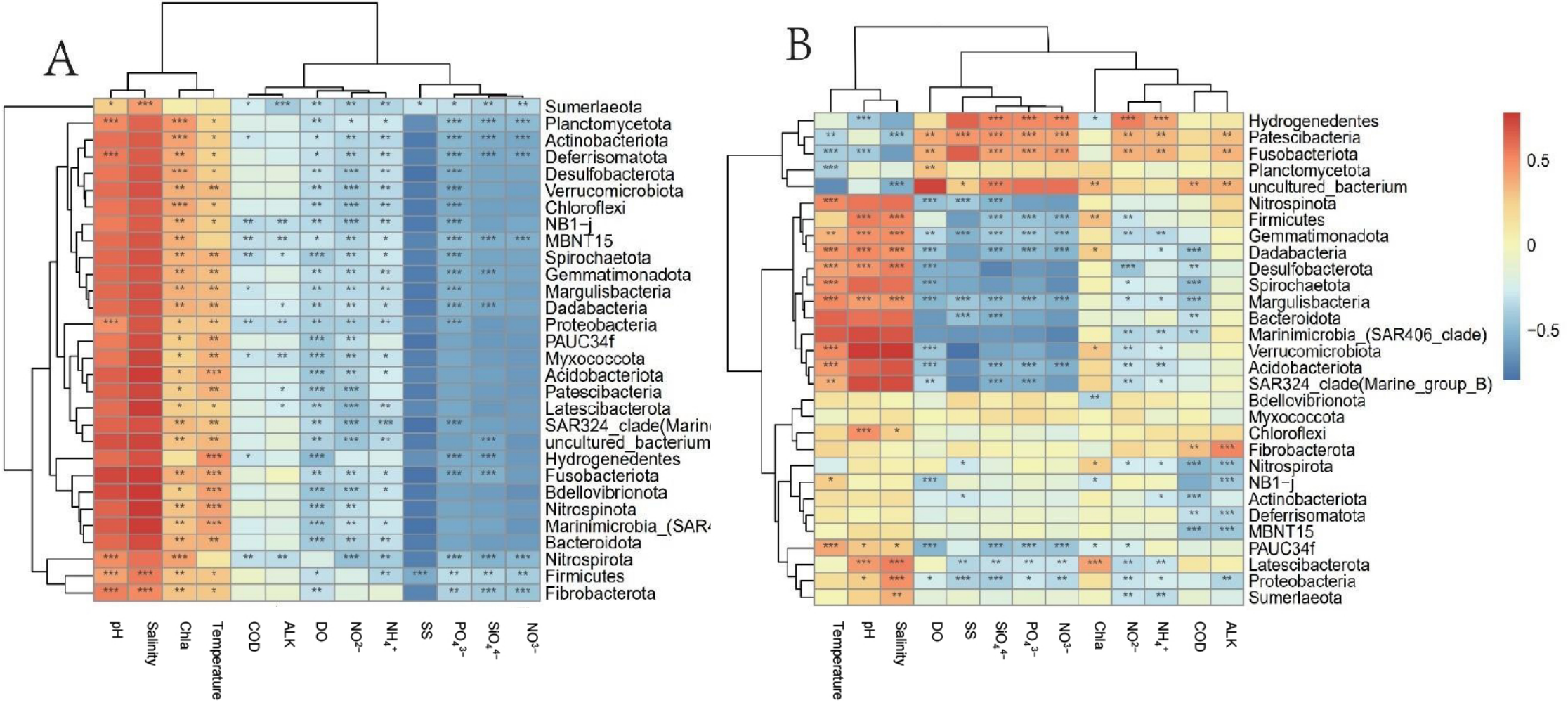

A
Sumerlaeota
Planctomycetota
Actinobacteriota
Deferrisomatota
Desulfobacterota
Verrucomicrobiota
Chloroflexi
NB1-j
MBNT15
Spirochaetota
Gemmatimonadota
Margulisbacteria
Dadabacteria
Proteobacteria
PAUC34f
Myxococcota
Acidobacteriota
Patescibacteria
Latescibacterota
SAR324_clade(Marin
uncultured_bacterium
Hydrogenedentes
Fusobacteriota
Bdellovibrionota
Nitrospinota
Marinimicrobia_(SAR4
Bacteroidota
Nitrospirota
Firmicutes
Fibrobacterota
pH
Salinity
Chla
Temperature
COD
ALK
DO
NO^{2-}
NH_4^+
SS
PO_4^{3-}
SiO_4^{4-}
NO^{3-}
B
Hydrogenedentes
Patescibacteria
Fusobacteriota
Planctomycetota
uncultured_bacterium
Nitrospinota
Firmicutes
Gemmatimonadota
Dadabacteria
Desulfobacterota
Spirochaetota
Margulisbacteria
Bacteroidota
Marinimicrobia_(SAR406_clade)
Verrucomicrobiota
Acidobacteriota
SAR324_clade(Marine_group_B)
Bdellovibrionota
Myxococcota
Chloroflexi
Fibrobacterota
Nitrospirota
NB1-j
Actinobacteriota
Deferrisomatota
MBNT15
PAUC34f
Latescibacterota
Proteobacteria
Sumerlaeota
Temperature
pH
Salinity
DO
SS
SiO_4^{4-}
PO_4^{3-}
NO^{3-}
Chla
NO^{2-}
NH_4^+
COD
ALK
0.5
0
−0.5
