# Supplementary material for: Distinct Assembly Processes Structure Planktonic Bacterial Communities Among Near- and Offshore Ecosystems in the Yangtze River Estuary
Source: Microb Ecol. 2024 Feb 14;87:42. doi: 10.1007/s00248-024-02350-x (PMC11385042; doi:10.1007/s00248-024-02350-x)
Supplement: Supplementary file 8 [file 248_2024_2350_MOESM8_ESM.pdf]

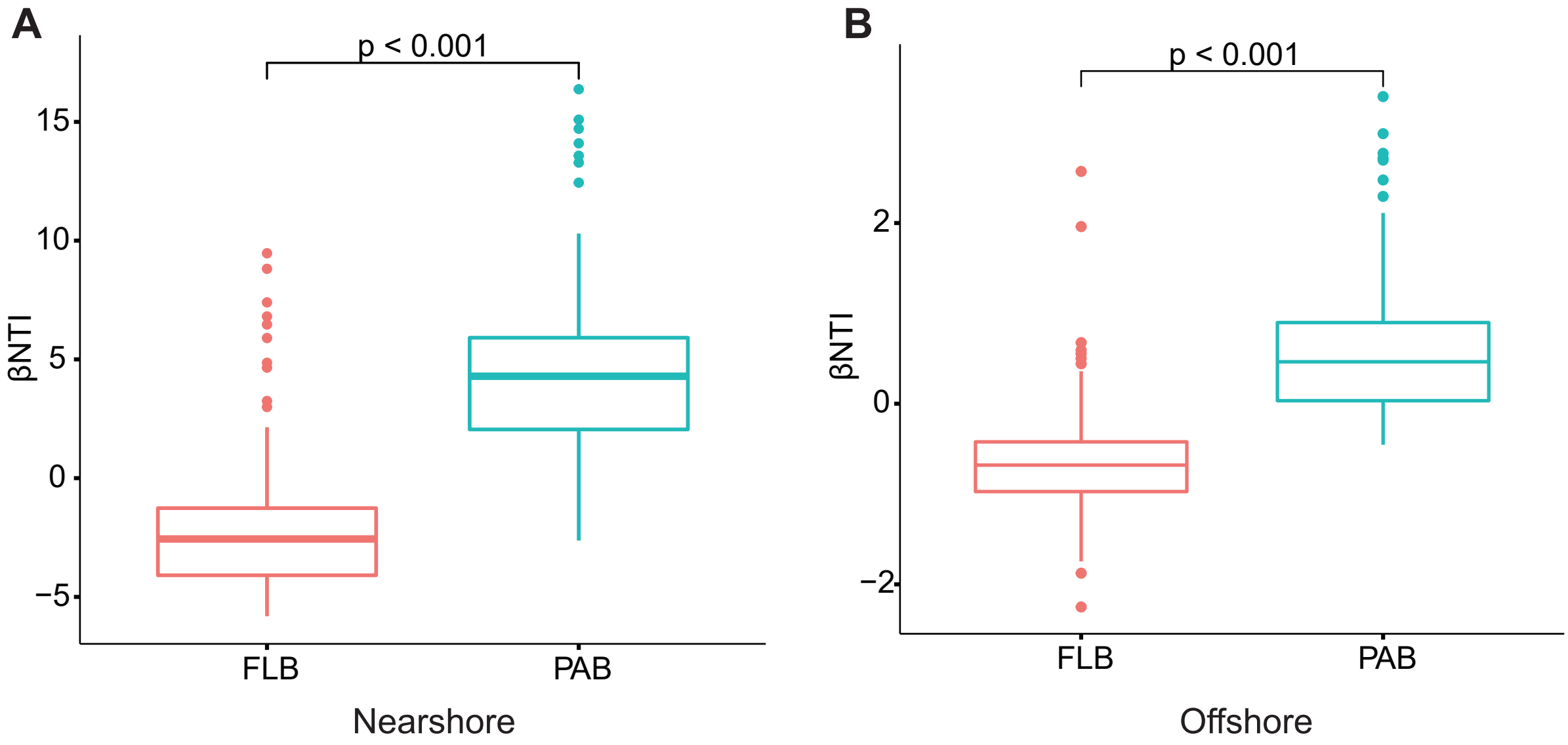

A
p < 0.001
15
10
5
0
−5
βNTI
FLB
PAB
Nearshore
B
p < 0.001
2
0
−2
βNTI
FLB
PAB
Offshore
