# Supplementary figures and images for: Distinct Assembly Processes Structure Planktonic Bacterial Communities Among Near- and Offshore Ecosystems in the Yangtze River Estuary
Source: Microb Ecol. 2024 Feb 14;87(1):42. doi: 10.1007/s00248-024-02350-x (PMC11385042; doi:10.1007/s00248-024-02350-x)

Rarefied Nounber of ASVs

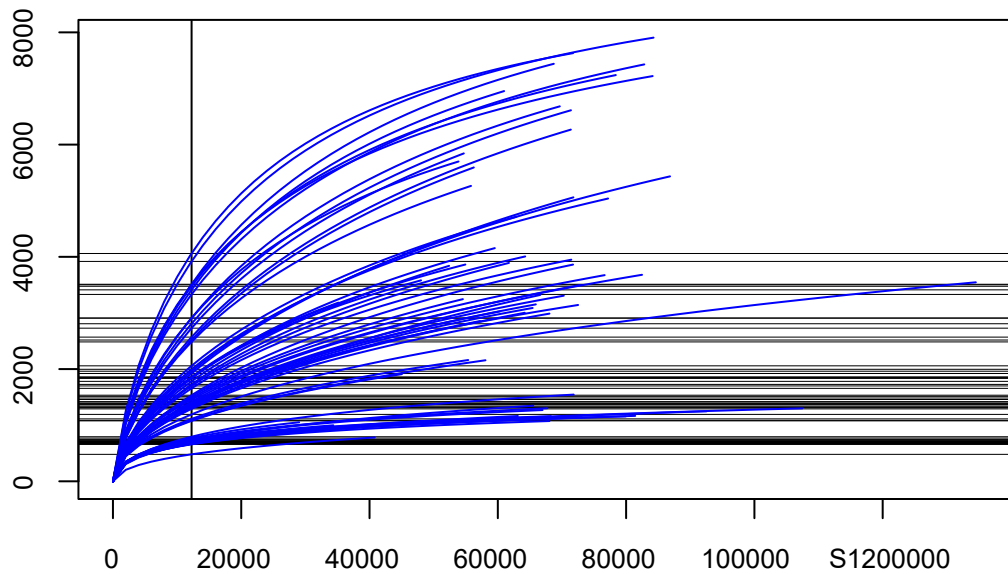

Supplement: Supplementary file 1 — (PDF 128 kb) [file 248_2024_2350_MOESM1_ESM.pdf]

**A**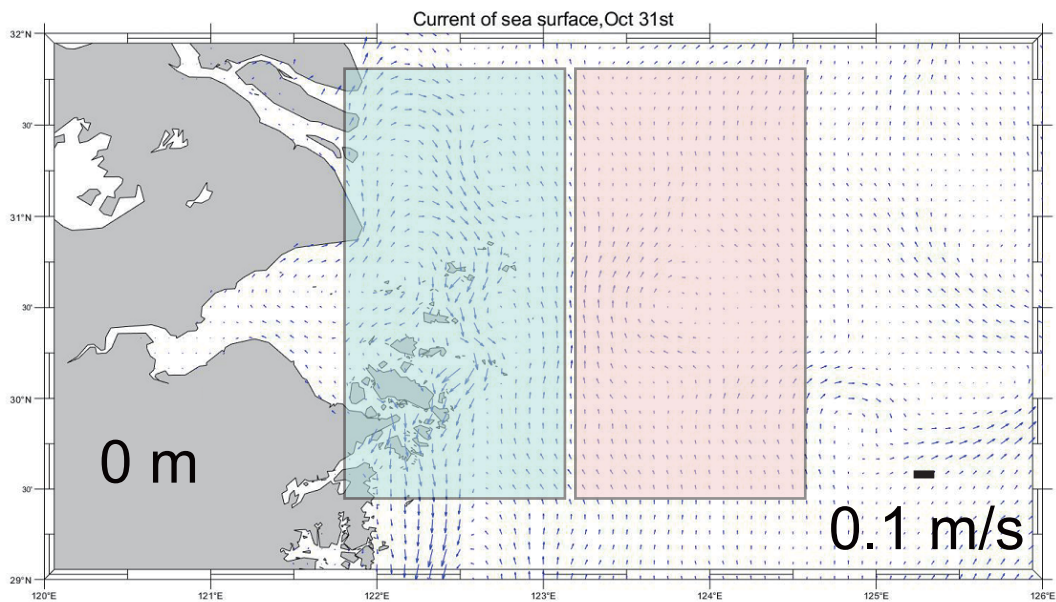**B**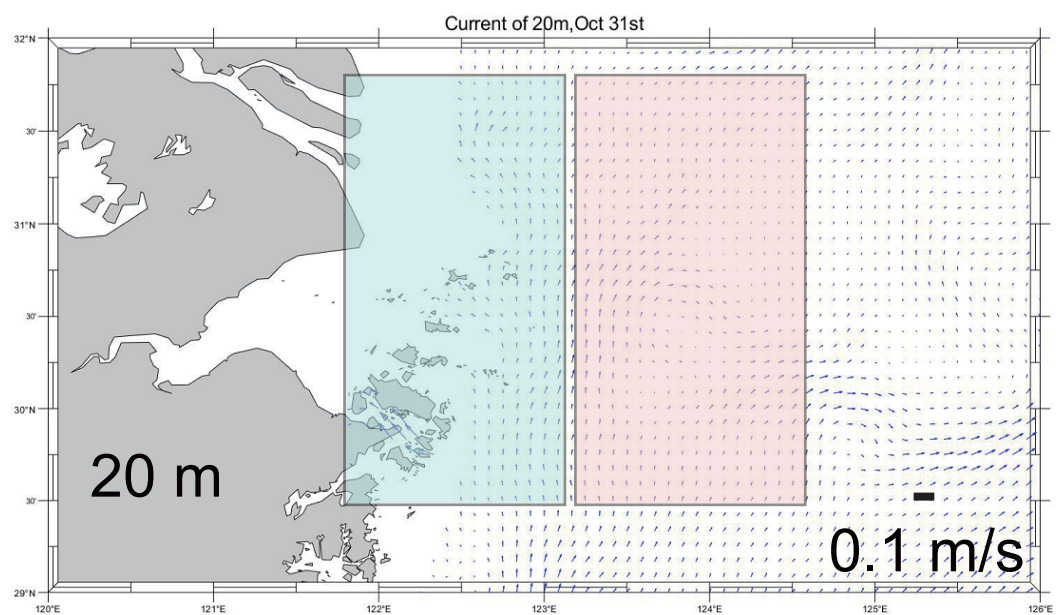**C**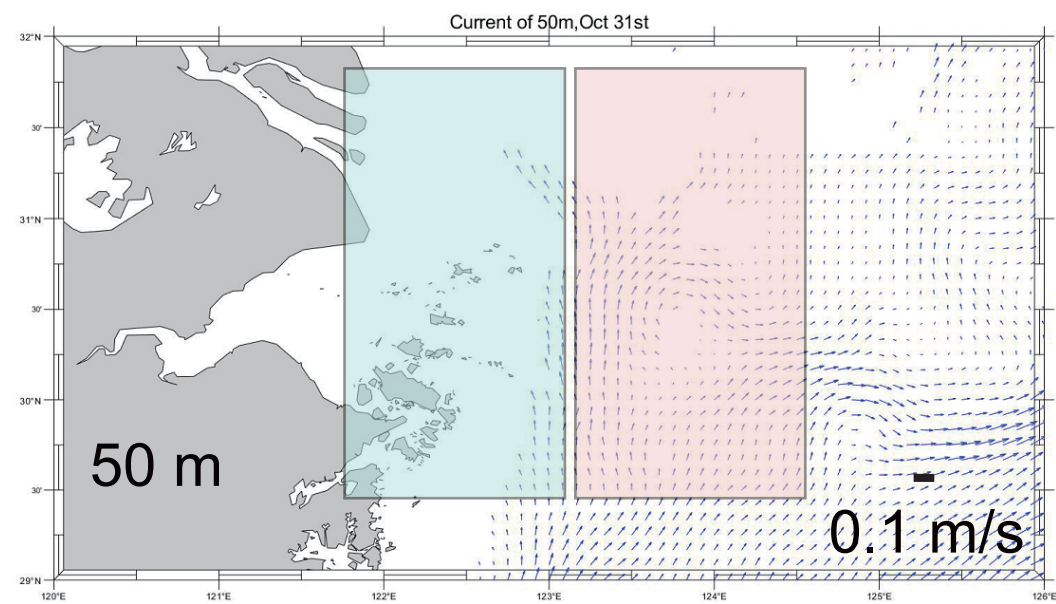

Supplement: Supplementary file 2 — (PDF 4424 kb) [file 248_2024_2350_MOESM2_ESM.pdf]

A

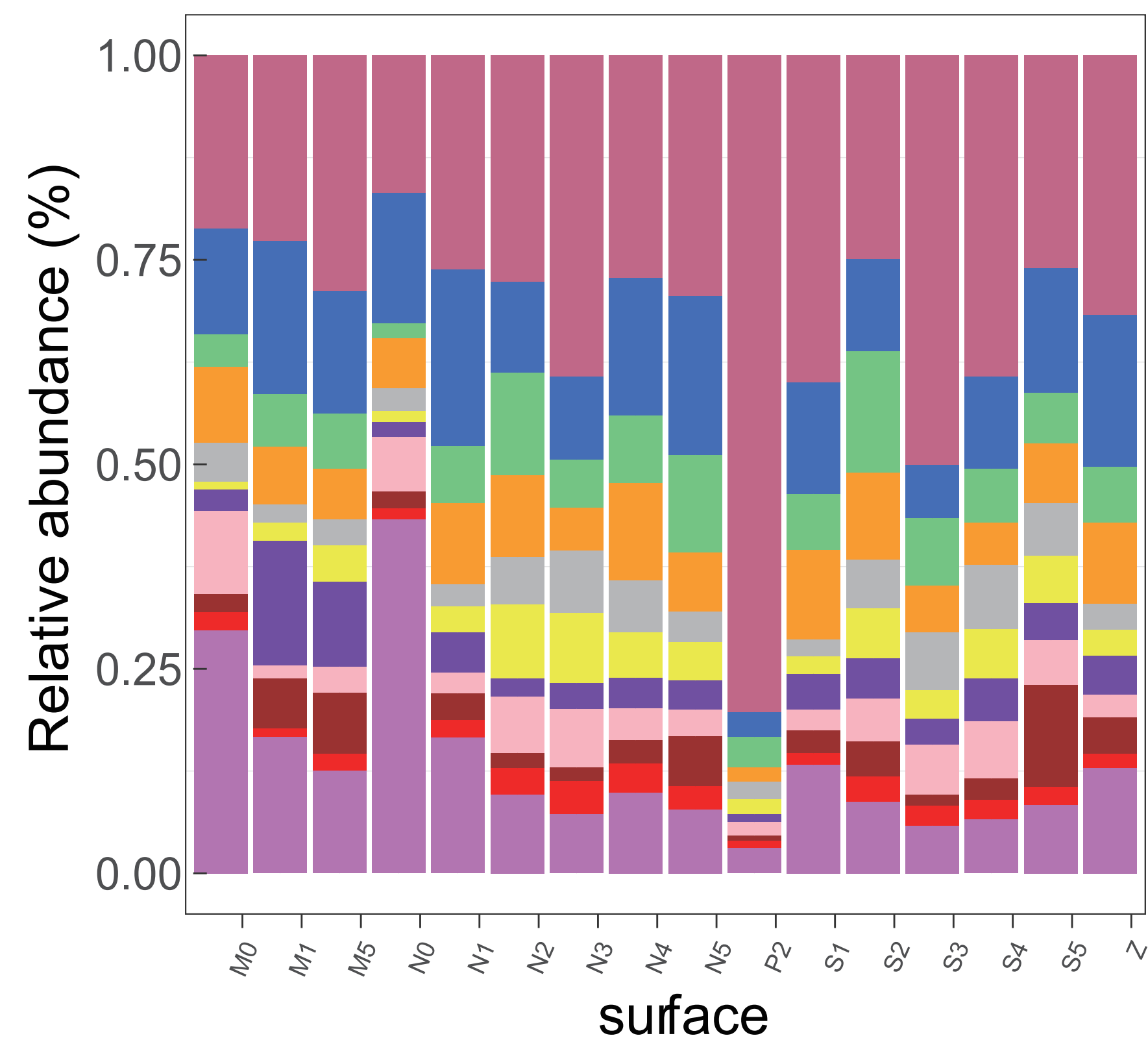

B

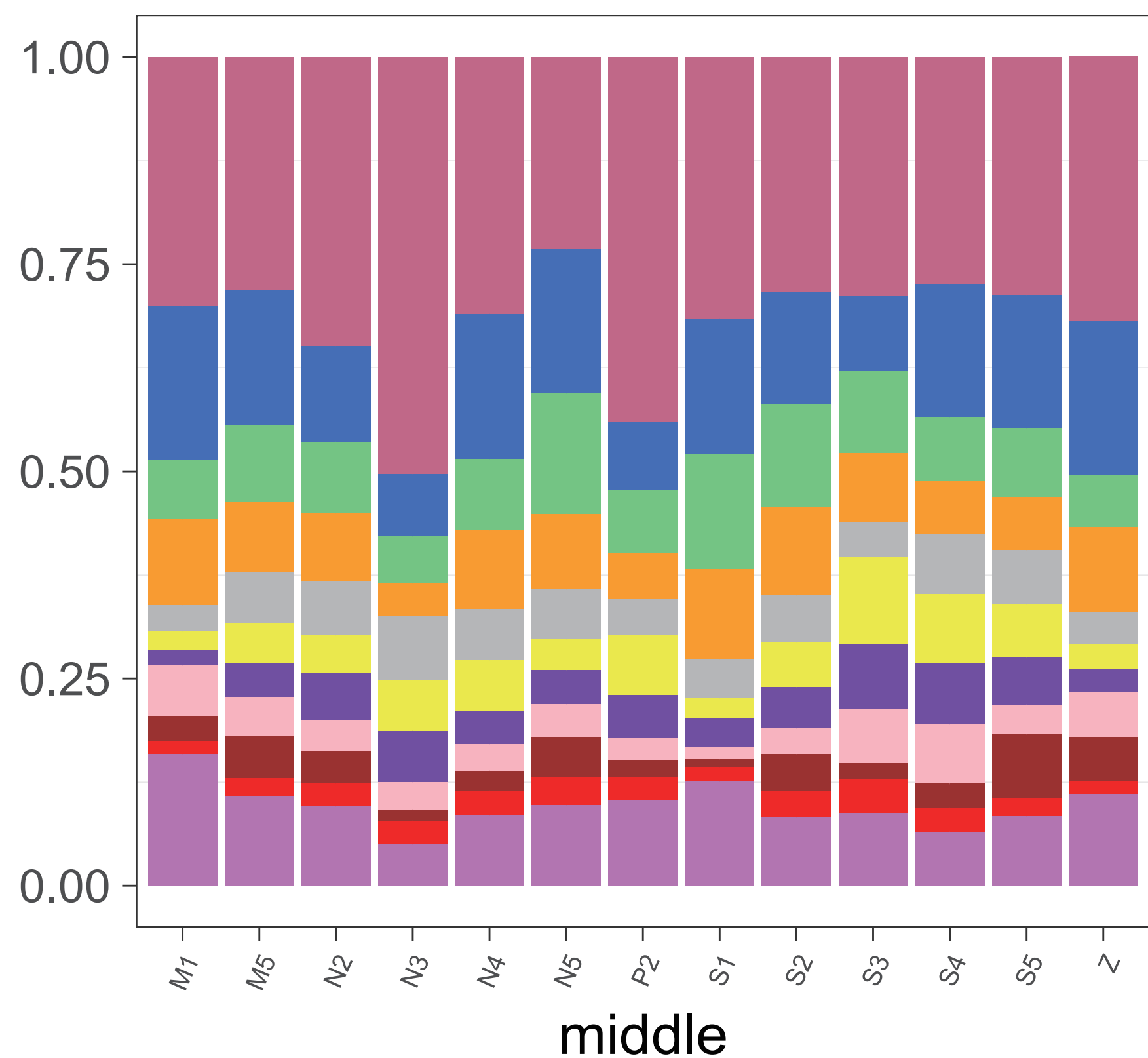

C

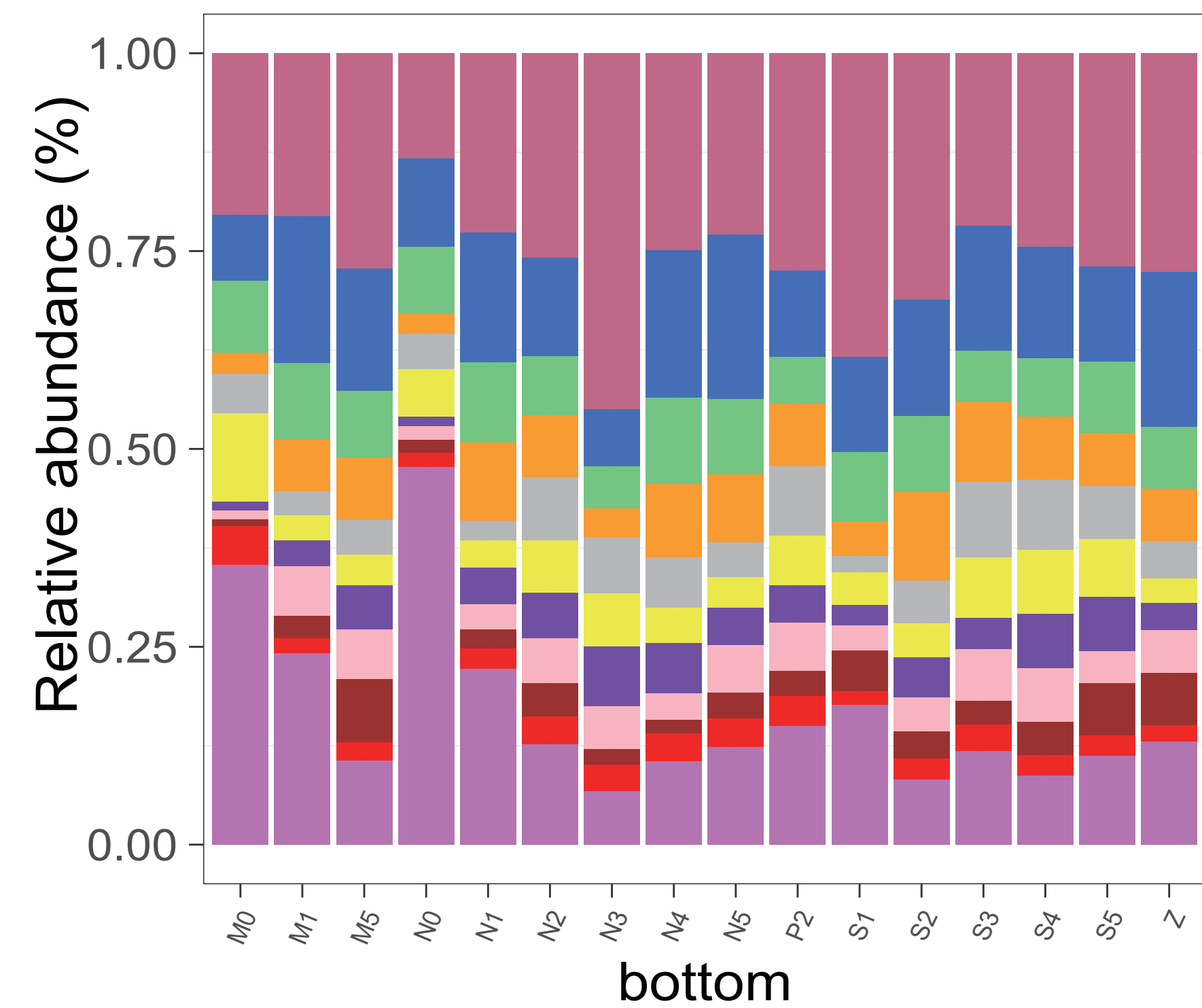

Group

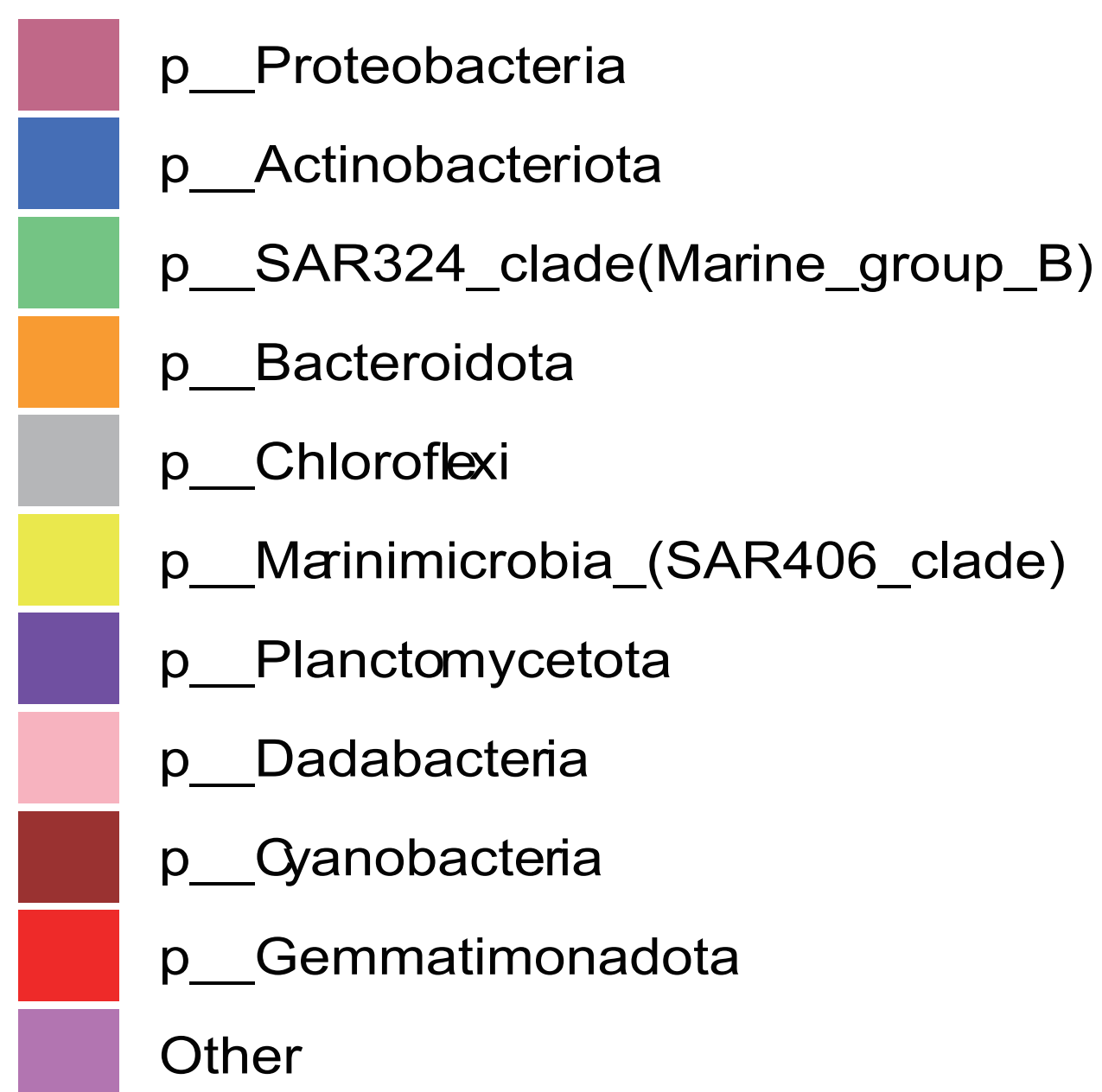

Supplement: Supplementary file 3 — (PDF 214 kb) [file 248_2024_2350_MOESM3_ESM.pdf]

## Nearshore

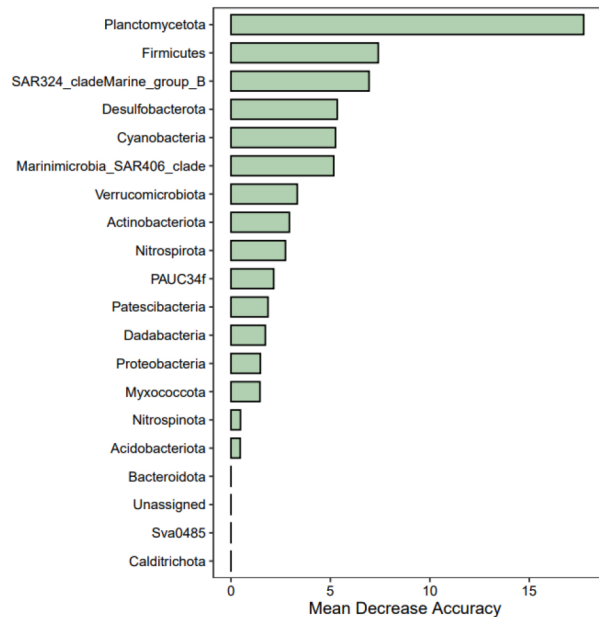

## Offshore

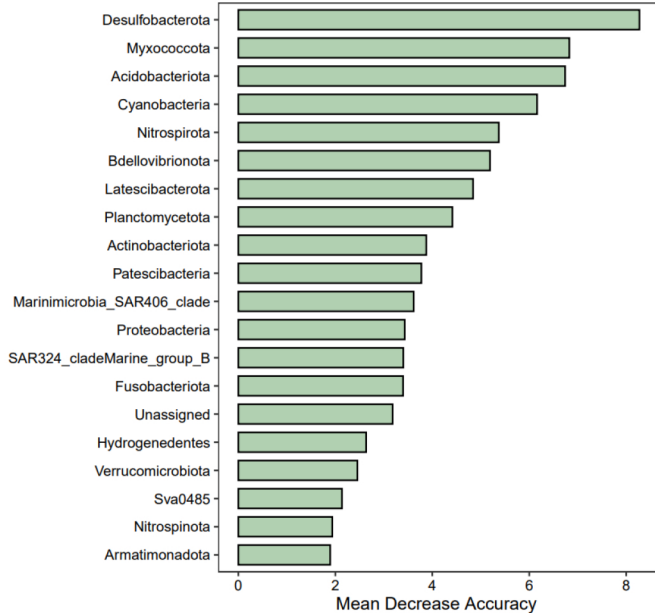

Supplement: Supplementary file 4 — (PDF 1312 kb) [file 248_2024_2350_MOESM4_ESM.pdf]

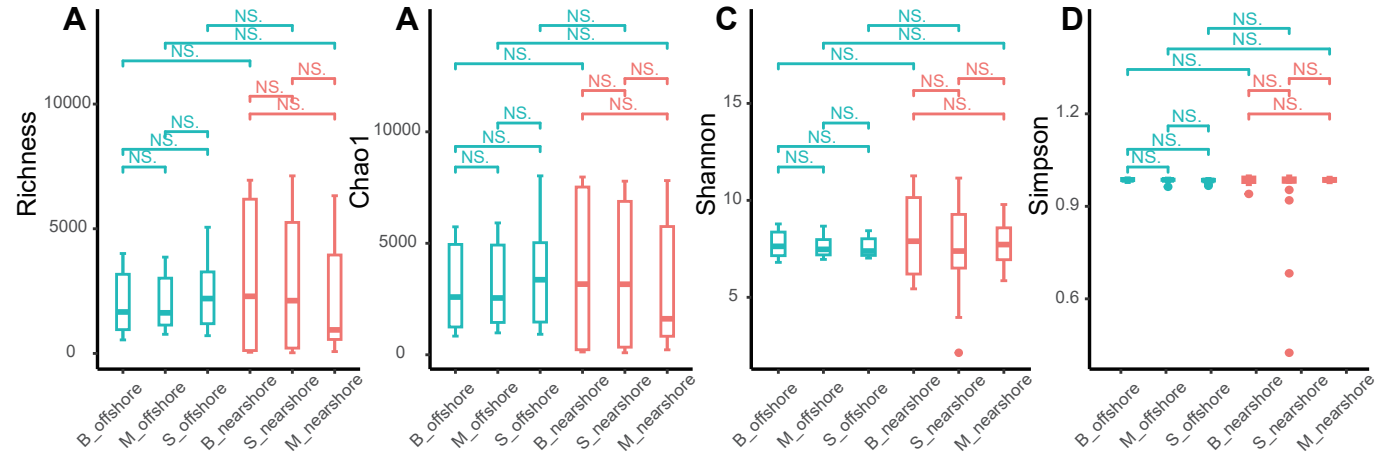

Supplement: Supplementary file 5 — (PDF 391 kb) [file 248_2024_2350_MOESM5_ESM.pdf]

## Nearshore

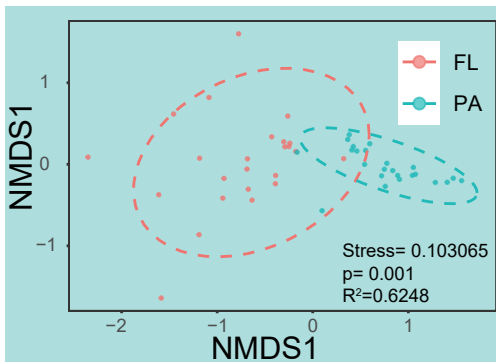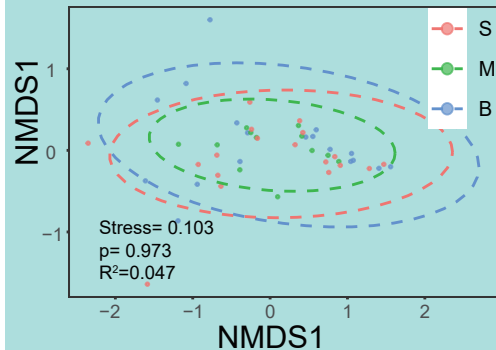

## Offshore

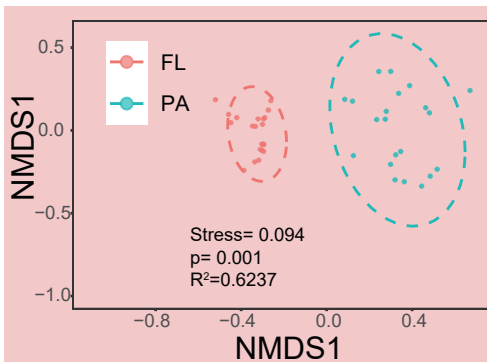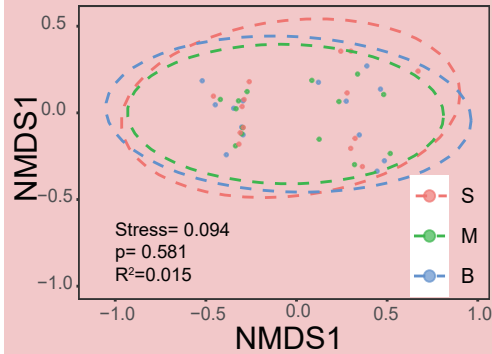

Supplement: Supplementary file 6 — (PDF 691 kb) [file 248_2024_2350_MOESM6_ESM.pdf]

A

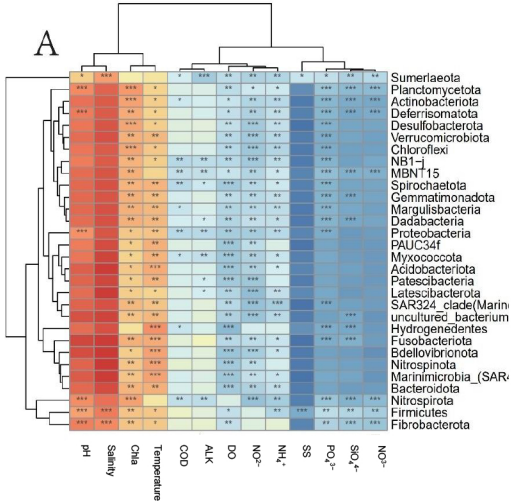

B

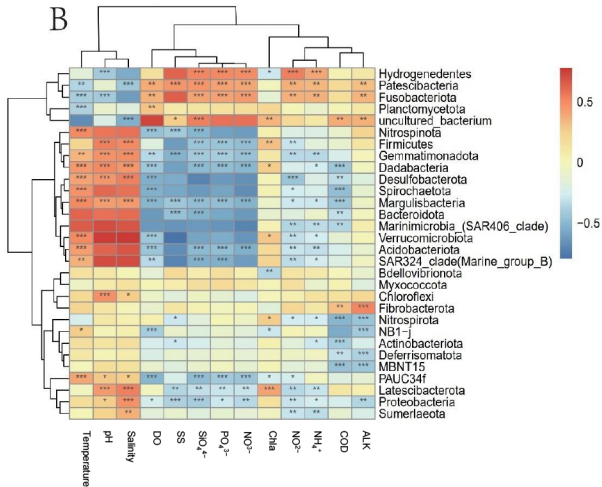

Supplement: Supplementary file 7 — (PDF 833 kb) [file 248_2024_2350_MOESM7_ESM.pdf]

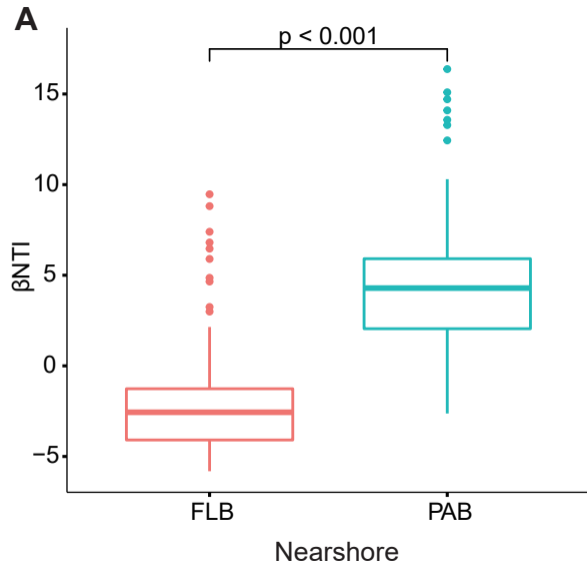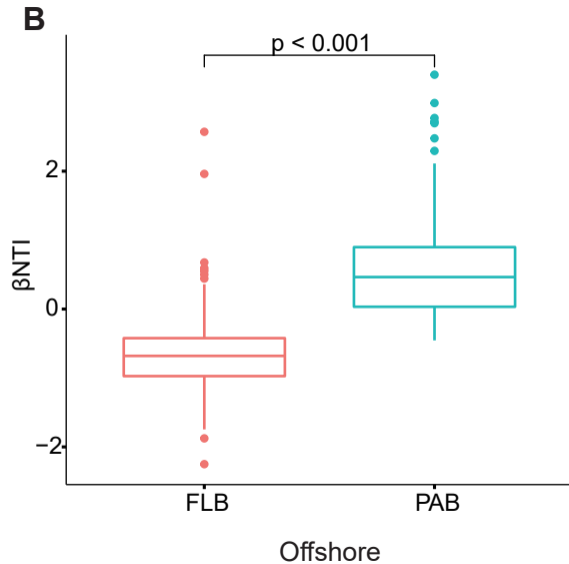

Supplement: Supplementary file 8 — (PDF 343 kb) [file 248_2024_2350_MOESM8_ESM.pdf]
